# *Keap1* Deletion Rescues Cell Death Associated With *Gpx4* Loss in Hepatocytes During Acute Liver Injury

**DOI:** 10.1111/liv.70210

**Published:** 2025-08-22

**Authors:** Leticia Colyn, Julia Grube, Chaochao Wang, Jana Dietrich, Mark Kühnel, Jörg Reinders, Karolina Edlund, Danny Jonigk, Nikolaus Gaßler, Jan Hengstler, Christian Trautwein

**Affiliations:** ^1^ Department of Internal Medicine III RWTH Aachen University Aachen Germany; ^2^ Institute of Pathology RWTH Aachen University Aachen Germany; ^3^ Leibniz Research Centre for Working Environment and Human Factors (IfADo) Dortmund Germany; ^4^ Member of the German Center for Lung Research (DZL) Biomedical Research in Endstage and Obstructive Lund Disease Hannover (BREATH) Hannover Germany; ^5^ Institute of Forensic Medicine, Section Pathology University Hospital of Jena Jena Germany

**Keywords:** acute liver failure, bile duct ligation, carbon tetrachloride, glutathione peroxidase 4, nuclear factor erythroid‐2 like

## Abstract

**Background & Aims:**

Acute liver failure (ALF) is a life‐threatening condition with limited treatment options beyond liver transplantation in non‐acetaminophen cases. The extensive loss of liver function results from severe hepatocyte death, where elevated reactive oxygen species (ROS) play a significant role. Nuclear factor erythroid‐2 like 2 (Nrf2) is crucial in ROS defence by regulating genes like glutathione peroxidase 4 (GPX4), which prevents lipid peroxidation (LPO). GPX4 is involved in several regulated cell processes, including apoptosis and ferroptosis.

**Methods:**

GPX4 expression was measured in liver samples from healthy, ALF, and acute‐on‐chronic liver failure (ACLF) patients. To investigate GPX4's role, mice with hepatocyte‐specific deletion of *Gpx4* (*Gpx4* ^Δhepa^) and both *Gpx4* and the Nrf2 repressor, *Keap1,* (*Gpx4* ^*Δ*hepa^ *Keap1* ^Δhepa^) were generated. ALF was induced in mice using carbon tetrachloride (CCl_4_) and bile duct ligation (BDL) cholestasis models, each lasting 48 h.

**Results:**

ALF patients exhibited reduced GPX4 levels compared to healthy individuals and ACLF patients, consistent with observations in CCl_4_‐treated wild‐type mice. ALF‐induced *Gpx4* ^Δhepa^ mice exhibited increased hepatocyte death and liver dysfunction upon CCl_4_, with increased apoptosis despite no changes in LPO markers. Activation of *Nrf2* in *Gpx4* ^Δhepa^ *Keap1* ^Δhepa^ mice reversed CCl_4_‐induced damage, reducing necrosis and apoptosis markers while inducing anti‐apoptotic BCL2.

**Conclusion:**

Our results demonstrate that *Gpx4* plays a critical role in ALF as its absence exacerbates apoptosis. Activating Keap1‐dependent pathways targeting antioxidant defence systems and upregulating BCL2 provides substantial protection against ALF in mice lacking *Gpx4* in hepatocytes. Our findings suggest that the Keap1‐Nrf2 axis is a promising therapeutic target in ALF.

**Abbreviations**
4HNE: 4‐hydroxynonenal
ACLF: acute‐on‐chronic liver failure
ACSL4: acyl‐CoA synthetase long chain family member 4
ALF: acute liver failure
ALI: acute liver injury
ALT: alanine transaminase
APAP: acetaminophen
AST: aspartate transaminase
BAD: BCL2 associated agonist of cell death
BAX: BCL2 associated X apoptosis regulator
BCL2: BCL2 apoptosis regulator
BDL: biliary duct ligation
CCl_4_
: carbon tetrachloride
CD163: CD163 molecule
CYP2E1: cytochrome P450 family 2 subfamily E member 1
DEGs: differentially expressed genes
DILI: drug‐induced liver injury
F4/80: F4/80 glycoprotein
FFPE: formalin‐fixed paraffin‐embedded
γH2AX: gamma H2A.X variant histone
GSDMD: Gasdermin D
GO: gene ontology
GCLC: glutamate‐cysteine ligase catalytic subunit
GSH: glutathione
GSSG: glutathione disulfide
GSR: glutathione‐disulfide reductase
GPX: glutathione peroxidase
GSTA: glutathione peroxidase‐active
H&E: haematoxylin–eosin
IHC: immunohistochemistry
CD11b: integrin alpha M
IL10: interleukin 10
IL1B: interleukin 1b
IL6: interleukin 6
KEAP1: Kelch‐like ECH‐associated protein 1
MDA: malondialdehyde
MRC1: mannose receptor C‐type 1
CYTB: mitochondrially encoded cytochrome b
MT‐ND1: mitochondrially encoded NADH:ubiquinone oxidoreductase core subunit 1
MLKL: mixed lineage kinase domain like pseudokinase
NQO1: NAD(P)H:quinone dehydrogenase 1
NRF2: nuclear factor erythroid‐2 like 2
PGD: phosphogluconate dehydrogenase
PRDX: peroxiredoxin
RCD: regulated cell death
RIPK3: receptor interacting serine/threonine kinase 3
ROS: reactive oxygen species
SQSTM1: sequestesome‐1
TXN: thioredoxin
TNFα: tumour necrosis factor alpha

**Summary**

- Acute liver failure is a severe condition with limited therapeutic options.
- In this study, we found that levels of the antioxidant protein GPX4 were reduced in patients with acute liver failure and that mice lacking GPX4 in hepatocytes showed increased liver injury.
- However, activating the Nrf2 pathway in these mice reduced liver cell death and increased the anti‐apoptotic protein BCL2 levels, highlighting the therapeutic potential of targeting the Keap1–Nrf2 axis in acute liver failure.

## 1 Introduction

Acute liver injury (ALI) refers to a massive and rapid deterioration of liver function in the absence of underlying liver disease triggered by for example, toxins, drugs, or viruses. If unresolved, ALI can progress to acute liver failure (ALF), a life‐threatening event that can lead to multiple organ failure and death [1]. The pathophysiology of ALI varies depending on the etiological agent but is broadly characterised by the overproduction of reactive oxygen species (ROS), culminating in hepatocyte death and loss of liver function [2]. The imbalance in redox homeostasis manifests as damage to plasma membranes and membrane‐bound organelles, leading not only to cellular dysfunction but also to cell lysis. This event is associated with lipid peroxidation, one of the main consequences of free radicals [3].

The liver is the metabolic and detoxification axis of the organism. To maintain the redox balance the liver developed a multi‐layered defence system. The enzymatic antioxidant system has a complex regulation consisting of three phases. Phase I includes enzymes that directly oxidise xenobiotics, increasing intracellular levels of ROS. Then these products are conjugated by Phase II enzymes, and finally, Phase III enzymes are responsible for the excretion of the final products from cells [4]. The induction of most Phase II enzymes is regulated by nuclear factor erythroid‐2 like 2 (Nrf2), including glutathione peroxidases (Gpx), NAD(P)H:quinone dehydrogenase1 (Nqo1), phosphogluconate dehydrogenase (Pgd), peroxiredoxin (Prdx), and thioredoxin (Txn). ROS accumulation causes NRF2 to disassociate from its repressor, Kelch‐like ECH‐associated protein 1 (KEAP1), allowing it to stabilise and translocate to the nucleus, where it initiates the antioxidant response [5].

Among GPX family members, isoform 4 (GPX4) is specialised to combat lipid peroxidation and maintain cell membrane integrity, making it one of the most important free radical scavenging antioxidant enzymes. Global deletion of Gpx4 causes embryonic death in mice, while conditional deletion in mice shows developmental defects, increased susceptibility to disease, and increased cell death. Thus, GPX4 deficiency has been found to be associated with multiple regulated cell death (RCD) mechanisms, including apoptosis, necroptosis, pyroptosis, and especially ferroptosis [6].

Experimental studies on ALI have shown that different RCDs are involved in hepatocyte death [7]. Since redox imbalance is a hallmark of hepatocyte death during ALI, in vivo models that mimic this situation are well established [8]. For instance, the administration of carbon tetrachloride (CCl_4_) has been used to study acute cases of liver failure, in which the trichloromethyl radical (•CCl_3_) causes the direct oxidation of macromolecules—lipids, proteins, and DNA—and triggers cell death [3].

In the present study, we investigated the function of GPX4 as an endogenous antioxidant in ALF. To this end, we evaluated GPX4 liver levels in patient samples and animal models of acute injury. The modulation of GPX4 levels in the disease led us to hypothesise that the absence of GPX4 would increase susceptibility to ferroptosis‐mediated cell death, as has already been shown [9]. To this end, we deleted GPX4 in hepatocytes and induced oxidative stress‐induced damage, followed by cell death. However, instead of the expected increase in lipid peroxidation, a hallmark of ferroptosis, we observed an increase in apoptosis. Since GPX4 belongs to the Phase II antioxidant defence, we speculated that activating the NRF2‐dependent antioxidant system might protect against oxidative damage associated with GPX4 deficiency in hepatocytes.

## 2 Methods

### 2.1 Human Samples

Formalin‐fixed paraffin‐embedded (FFPE) liver samples from patients with acute liver failure (ALF, *n* = 16) or acute‐on‐chronic liver failure (ACLF, *n* = 14) undergoing transplantation between 2013 and 2023 were retrospectively collected from the RWTH University Hospital pathology archive. See Table S1 for a detailed description of patients with liver failure etiologies. Samples without pathological changes associated with liver disease (*n* = 5) were used as healthy controls. The use of pseudonymized FFPE samples was approved by the Ethics Committee of the Medical Faculty of RWTH Aachen University (EK23‐350).

### 2.2 Mouse Generation and Animal Experiments

Hepatocyte‐specific *Gpx4* and *Keap1* knockout mice were generated by crossing a C57BL/6 *Alfp‐Cre* transgenic mouse strain [10] with floxed *Gpx4* (*Gpx4* ^f/f^) and *Keap1* (*Keap1* ^f/f^) mice, respectively. We crossed mice with hepatocyte deletion of *Gpx4* (*Gpx4* ^Δhepa^) to mice with hepatocyte deletion of *Keap1* (*Keap1* ^Δhepa^) to generate mice with dual deletion of *Gpx4* and *Keap1* in hepatocytes (*Gpx4* ^Δhepa^ *Keap1* ^Δhepa^). *Alfp‐Cre*‐negative littermates served as controls (*Gpx4* ^f/f^). Mice were housed under specific pathogen‐free conditions, with a 12‐h light/dark cycle and access to autoclaved chow and sterilised water. *Gpx4* knockout mice were supplemented with vitamin E (S0382‐S043; Ssniff Spezialdiaeten GmbH) throughout gestation and lactation (adapted from Carlson et al. [11]) as approved by the animal protection authorities (Authority for Environment Conservation and Consumer Protection of the State of North Rhine‐Westphalia, LANUV, Recklinghausen, Germany; file ref. AZ‐84‐02.04.2019.A490).

Acute liver injury was induced in 8‐week‐old male mice using a single intraperitoneal injection (i.p.) of carbon tetrachloride (CCl_4_, 0.6 mL/kg; Sigma‐Aldrich) diluted in corn oil [12]. After 24 or 48 h, mice were sacrificed, and tissues were collected and stored for further analysis. Biliary duct ligation (BDL) and sham surgery were performed as described previously [13], with mice sacrificed at 48 h post‐surgery. All animal experiments were performed under approvals from the appropriate authorities for animal welfare (LANUV file ref. AZ‐84‐02.04.2016.A080 and AZ‐81‐02.04.2020.A390) and in compliance with EU Directive 2010/63/EU.

### 2.3 Histology and Immunohistochemistry

For human FFPE samples, immunohistochemistry (IHC) was performed using the EnVision Flex High pH detection kit (K8000, Dako), with 2 μm sections undergoing antigen retrieval in Tris/EDTA buffer (pH 9) at 95°C for 20 min. Endogenous peroxidase activity was blocked with peroxidase‐blocking Flex solution for 15 min, and sections were incubated with GPX4 antibody for 30 min. After washing, the slides were incubated with a dextran polymer conjugated to secondary antibodies and horseradish peroxidase (HRP) for a further 20 min and stained with DAB for 10 min. Sections were counterstained with Mayer's haematoxylin and mounted.

For murine samples, livers were fixed in 4% paraformaldehyde for 12 h, stored in 70% ethanol, and embedded in paraffin. Sections were cut with a thickness of 3 μm. FFPE sections were stained with haematoxylin–eosin (H&E). IHC for GPX4, 4HNE, and cleaved caspase‐3 was performed following antigen retrieval using 10 mM sodium citrate (pH 6.0) for 10 min and 3% H_2_O_2_ for 10 min, and blocking using a peroxidase‐conjugated polymer method (MP‐7451, Vector Laboratories). For primary antibodies used see Table S2. Visualisation was achieved using ImmPACT DAB peroxidase substrate (SK4105; Vector Laboratories), followed by haematoxylin counterstaining (MHS32; Merck Millipore). Finally, the sections were dehydrated and mounted with Roti‐Histokitt (6638.1; Carl Roth).

For immunofluorescence, cryosections were fixed in 4% PFA and blocked with DPBS + 0.02% NaAzid + 0.2% BSA. The sections were then incubated with the primary antibody CD11b (ab8878; 1:200 dilution; Abcam) diluted in 1% mouse serum. After washing with DPBS + 0.02% NaAzid, the sections were incubated with the secondary antibody goat anti‐rat/Cy3 (A10522; 1:500 dilution; Invitrogen) in 1% mouse serum for 1 h. After washing and re‐blocking with DPBS + 0.02% NaAzid + 0.2% BSA, the sections were incubated with the primary antibody F4/80 (MCA497; 1:200 dilution; Bio‐Rad Laboratories). The next day, the sections were washed and incubated with the secondary antibody goat anti‐rat/Alexa 488 (A‐11006; 1:500 dilution; Invitrogen) in 1% mouse serum. Finally, the sections were counterstained with a DAPI‐containing mounting medium (H‐1200; Vector Laboratories).

Apoptosis was detected using the TUNEL assay (11 684 795 910, Merck Millipore) on 5 μm sections, with staining performed per the manufacturer's instructions. Sections were counterstained with DAPI‐containing mounting medium.

GPX4 quantification for IHC of human samples was done blinded by expert pathologists using an Olympus BX51 microscope (Olympus Life Science). Briefly, hot spot regions of each sample were placed at 400× magnification, and cells showing intense and granular cytoplasmic staining were manually counted. Quantification is expressed as a percentage of positive cells relative to total cells in the field. A total of three different fields of view were analysed for each sample. For murine samples, quantification was done with the Axio Imager Z1 (Carl Zeiss) for brightfield images or the Axio Imager A2 (Carl Zeiss) for fluorescence images and processed with the axioVision LE64 version 4.9.1 software (Carl Zeiss). H&E, 4HNE, and GPX4 images were captured at 10× magnification, and cleaved caspase‐3 and TUNEL were captured at 20× magnification. For cleaved caspase‐3 and TUNEL analysis, positive cells were counted manually. For H&E, 4HNE, and GPX4 analysis, the positively stained area was determined using ImageJ software (version 1.46; National Institute of Health), as previously described [14].

### 2.4 Immunoblot Analysis

Frozen liver tissues were homogenised in NP40 lysis buffer, and protein concentration was measured by the Bradford assay. Proteins were separated by SDS‐PAGE, transferred to nitrocellulose membranes, and probed with primary antibodies overnight (see Table S2). After washing, membranes were incubated with HRP‐conjugated secondary antibodies (Table S2). Labelled proteins were visualised using enhanced chemiluminescence (RPN2232, Merk Millipore), and the resulting light emissions were detected with an ImageQuant LAS 4000 fluorescence image analyser (GE Healthcare).

### 2.5 Flow Cytometry

First, capillary leukocytes were removed from the liver tissue by perfusion with PBS. Then, the liver tissue was digested at 37°C for 45 min using Collagenase type 4 (LS004189, Worthington). Afterwards, the liver was minced through a 70 μm cell strainer, and the remaining erythrocytes were lysed using BD Pharm Lyse buffer (555 899, BD). The resulting cell suspension was stained with fluorochrome‐conjugated antibodies (1:300) for myeloid cells or lymphoid cells for 30 min at 4°C (see Table S2), Calibrite APC beads (340487, BD), and Hoechst 33258 as a viability dye. Sample analysis was performed using an LSR Fortessa flow cytometer (BD) and FlowJo software (version 10.4.2, BD). Cells were pre‐gated as Hoechst‐CD45+ to identify viable leukocytes. Total cells per liver were calculated using Calibrite APC beads for calibration.

### 2.6 Biochemical Assays

Blood samples were collected from the inferior vena cava, and serum alanine transaminase (ALT) and aspartate transaminase (AST) were measured using standard procedures at the central laboratory of RWTH Aachen University Hospital.

Lipid peroxidation was assessed via 4HNE IHC and by measuring malondialdehyde (MDA) content using the Lipid Peroxidation (MDA) Assay Kit (ab118970, Abcam), following the manufacturer's protocol.

### 2.7 Glutathion Analyses by LC–MS/MS

Reduced and oxidised glutathione were analysed by LC‐triple quad‐MS in MRM mode as published previously [15]. Briefly, tissue samples were extracted using 100 μL 250 mM N‐ethyl‐maleimide, 1.5 mg/mL Na_2_‐EDTA 2 × H_2_O in H_2_O/methanol (3:2, v/v), pH 7.4 (adjusted using NaHCO_3_) and incubated for 15 min at room temperature. Proteins were precipitated with trichloroacetic acid and the samples were extracted with dichloromethane. The upper phase was lyophilised and reconstituted in 1 mL 0.1% formic acid. 5 μL were used for injection to the LC‐TQ‐MS operating in MRM mode. Quantification was accomplished using the Skyline software (version 24.1) [16].

### 2.8 RNA Isolation and RT‐qPCR

RNA was extracted from frozen liver tissue using TRIzol reagent (15596026; Thermo Fisher Scientific), and cDNA was synthesised from 2 μg RNA using the High‐Capacity cDNA Reverse Transcriptase Kit (4368813; Applied Biosystems). RT‐qPCR was performed using SYBR Green Master Mix (A25778; Thermo Fisher Scientific) on a QuantStudio 5 Real‐Time PCR System (Thermo Fisher Scientific). Gene expression was normalised to *Gapdh* using the 2‐ΔΔCt method. The sequence of the primers used is shown in Table S3.

### 2.9 RNA Sequencing

RNA concentrations were determined on a Qubit 4 Fluorometer with the RNA BR Assay Kit (Thermo Fisher). RNA integrity was assessed on a 2100 Bioanalyzer with the RNA 6000 Nano Kit (Agilent Technologies). Sequencing libraries were generated from 300 ng RNA, using the Illumina Stranded mRNA Prep Ligation kit with unique dual indexes (Illumina), according to the manufacturer's protocol. Quantification of the libraries was performed with the Qubit 1X dsDNA HS Assay Kit (Thermo Fisher), and library sizes were checked on an Agilent 2100 Bioanalyzer with the DNA 1000 Kit (Agilent Technologies). The libraries were then normalised, pooled, diluted to 1.05 pM, and paired‐end sequenced (2 × 75 bp) using the 500/550 High Output Kit v2.5 (Illumina) on an Illumina NextSeq 550. Mapping and quantification of the FASTQ files was done using Salmon [17] and the option ‘partial alignment’ with the online provided decoy‐aware index for the mouse genome. To summarise the transcript reads on the gene level, the R package tximeta [18] was used. Genes with at least 10 counts across all mice were included in further analysis. The R package DeSeq2 [19] was used to calculate differentially expressed genes, with the option “ashr” to shrink the obtained fold changes. As a cutoff for differential expression, fdr adj *p* ≤ 0.05 and |log2FC| > log2(1.5) was applied. Gene Ontology (GO) term enrichment analysis was performed using the R package topGO [20]. R version 4.2.3 was used.

### 2.10 Statistical Analysis

Data were analysed using Prism software (version 9.4.1; GraphPad) and are depicted as mean values with error bars indicating standard error of the mean (SEM). For each experiment, the number of animals (*n*) and the statistical test are indicated in the figure legend. Normal distribution within each group was verified using the D'Agostino and Pearson normality test (for *n* ≥ 7) or the Shapiro–Wilk normality test (for *n* < 7). Comparisons between two groups were analysed by a two‐tailed unpaired t‐test (if both groups followed a normal distribution) or a Mann–Whitney test (if not normal‐distributed). Comparisons between more than two groups (with comparison of several conditions) were analysed using a one‐way ANOVA with Tukey's multiple comparisons test (if normal‐distributed) or a Kruskal–Wallis test with a Dunn's multiple comparisons test (if not normal‐distributed). Differences were considered significant when *p* values were below 0.05. The level of significance is indicated in each figure (i.e., **** *p* < 0.0001; *** *p* < 0.001; ** *p* < 0.01; * *p* < 0.05; ns, not significant).

## 3 Results

### 3.1 GPX4 Expression in Liver Failure

**FIGURE 1:**
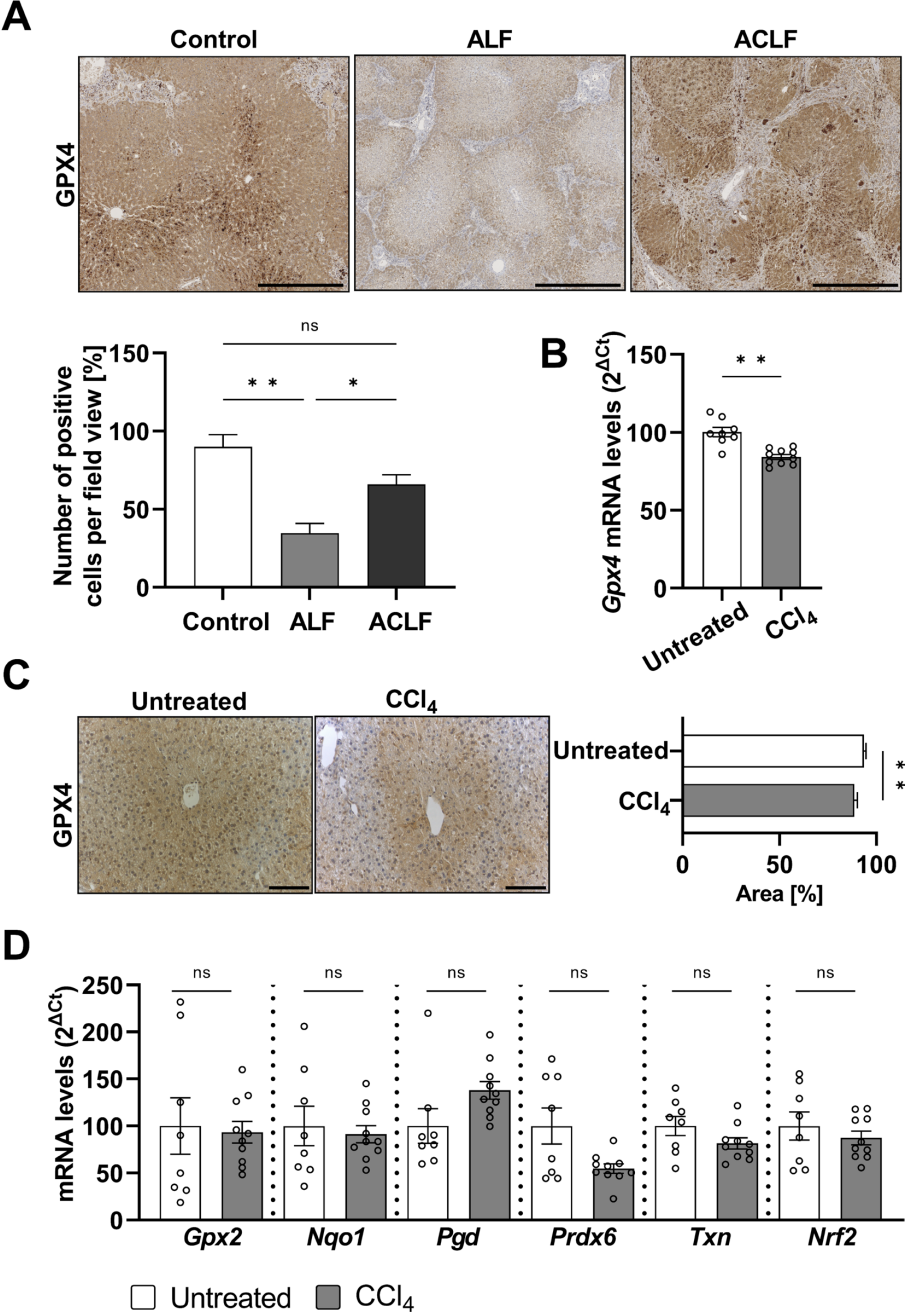
Antioxidant system regulation in liver failure. (A) GPX4 immunohistochemistry of human samples. Above: Representative images are shown, scale bar = 500 μm. Bottom: GPX4 positive cells quantification relative to the total number of cells per field view; control (*n* = 5), ALF (*n* = 16), ACLF (*n* = 14). (B) *Gpx4* mRNA expression in livers from control (*n* = 8) or CCl_4_‐treated (*n* = 10) WT mice. (C) GPX4 immunohistochemistry of murine livers. Left: Representative images are shown, scale bar = 100 μm. Right: GPX4 positive area relative to total area; untreated (*n* = 4), CCl_4_ (*n* = 6). (D) mRNA levels of phase II antioxidant enzymes expression in livers from control (*n* = 8) or CCl_4_‐treated (*n* = 10) WT mice. Data are expressed as ± SEM. Kruskal–Wallis test with Dunn's multiple comparison test was performed for panel A; unpaired *t* test comparing treated to untreated mice was conducted for panel (B–D). ns, not significant; *p < 0.05; **p < 0.01. Acute liver failure, ALF; acute‐on‐chronic liver failure, ACLF; glutathione peroxidase 2, Gpx2; NAD(P)H:Quinone dehydrogenase 1, Nqo1; nuclear factor erythroid‐2 like 2, Nrf2; phosphogluconate dehydrogenase, Pgd; peroxiredoxin 6, Prdx6; thioredoxin, Txn.

To understand the expression of GPX4 in normal liver and in livers derived from patients with acute liver injury, we performed IHC staining for GPX4 expression in liver tissue samples. One expert pathologist determined a decrease in GPX4 levels in hepatocytes close to the injury site in ALF patients compared to control individuals and acute‐on‐chronic liver failure (ACLF) patients (Figure 1A). Similarly, we determined GPX4 levels in mouse liver after inducing acute liver injury via CCl_4_ administration. Analysis of *Gpx4* mRNA levels showed that after injury, there was a decrease in its expression (Figure 1B) that was also confirmed on the protein level using IHC stainings (Figure 1C).

Since GPX4 is a phase II antioxidant enzyme, we evaluated the levels of other detoxifying enzymes, such as *Gpx2*, *Nqo1*, *Pgd*, *Prdx6*, and *Txn*, as well as *Nrf2* expression (Figure 1D). We did not observe any variation in the expression levels of phase II enzymes, except for *Gpx4*. These results suggest an essential role for GPX4 during the acute response to a hepatotoxic agent such as CCl_4_. Based on these experiments, we hypothesised that changes in *Gpx4* expression in hepatocytes might be relevant to determine the course of CCl_4_‐induced ALF.

**FIGURE 2:**
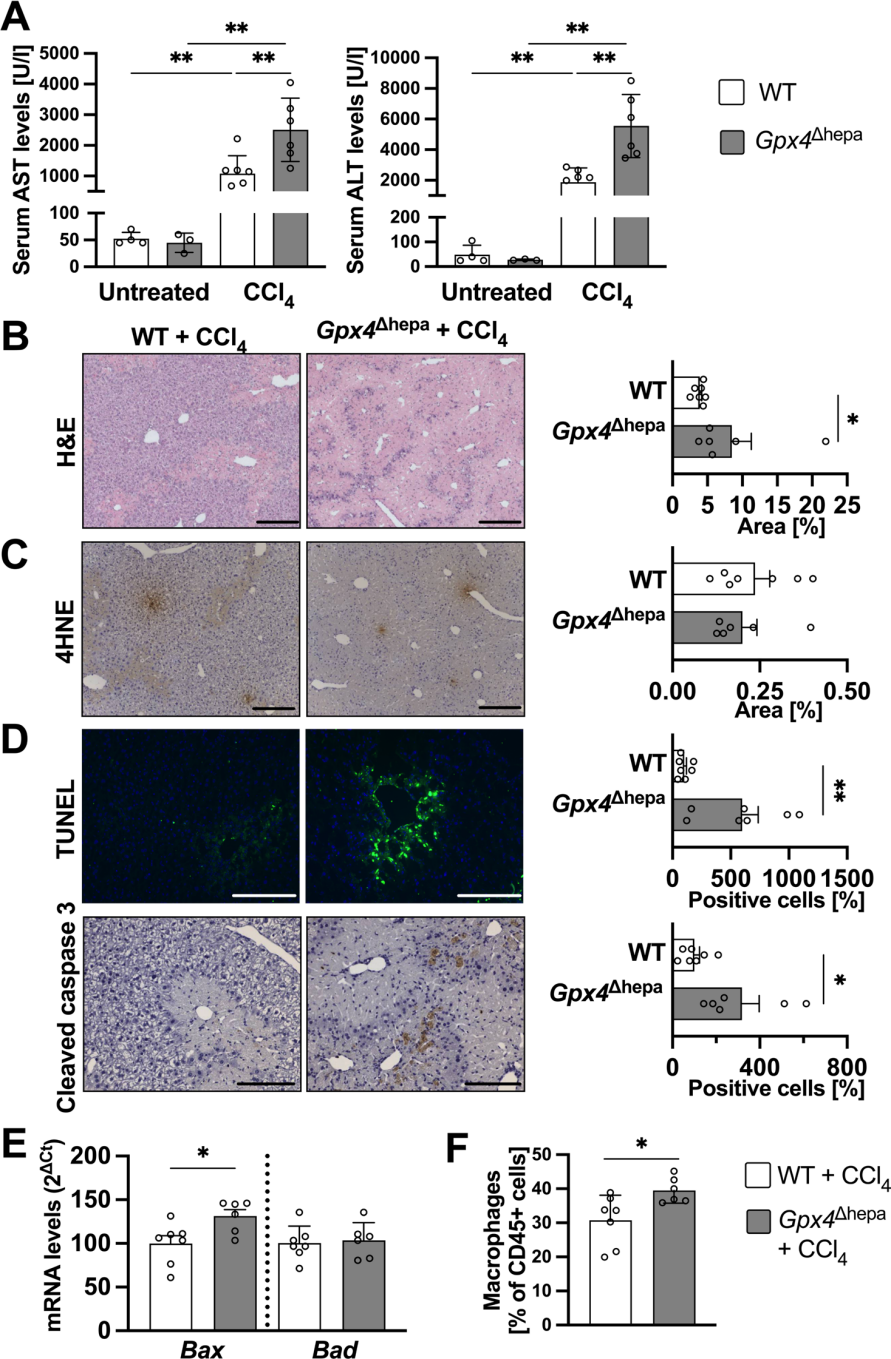
*Gpx4* deficiency in hepatocytes exacerbates liver injury and cell death. (A) Serum transaminases levels (left: AST, right: ALT) in untreated and CCl_4_‐treated WT (untreated, *n* = 4; treated, *n* = 7; white) and *Gpx4* ^Δhepa^ (untreated, *n* = 3, treated, *n* = 6; grey) mice. (B) Left: Haematoxylin–eosin stainings of liver sections from CCl_4_‐treated WT and *Gpx4* ^Δhepa^ mice. Scale bars = 100 μm. Right: Necrosis area ratios comparison among CCl_4_‐treated WT and *Gpx4* ^Δhepa^ mice. (C) 4‐hydroxynonenal IHC staining. Left: Representative images. Scale bar = 100 μm. Right: Percentage of positive area for 4HNE‐adducts. (D) Above: TUNEL staining. Left: Representative images. Scale bar = 200 μm. Right: Percentage of positive cells stained for TUNEL per view field relative to the WT group. Bottom: Cleaved caspase 3 IHC staining. Left: Representative images. Scale bar = 200 μm. Right: Percentage of positive cells stained for cleaved caspase 3 per view field relative to the WT group. (E) mRNA levels for pro‐apoptotic markers. (F) Infiltrating leukocytes were isolated from livers. Cells were stained for CD45 to identify leukocytes and with Hoechst 33258 to exclude dead cells. Cells were stained for suitable markers and gated as macrophages (CD11b^+^F4/80^+^). Data are expressed as ± SEM from CCl_4_‐treated WT (*n* = 7) and *Gpx4* ^Δhepa^ (*n* = 6). Ordinary one‐way analysis of variance (ANOVA) with Tukey's multiple comparison test was performed for panel A; unpaired t test comparing *Gpx4* ^Δhepa^ to WT mice was conducted for panel (B–F). *p < 0.05; ** < 0.01. 4‐hydroxynonenal, 4HNE; aspartate transaminase, AST; alanine transaminase, ALT; BCL2 associated X apoptosis regulator, Bax; BCL2 associated agonist of cell death, Bad; haematoxylin–eosin, H&E.

Based on these results and to functionally define the role of GPX4 in hepatocytes in situations leading to liver failure, we generated mice with a hepatocyte‐specific deletion of *Gpx4* (Figure S1) and used the acute CCl_4_ model to induce ALF. 48 h after CCl_4_ administration, we observed an exacerbation of markers of hepatocellular injury and impaired function in the *Gpx4*‐deficient group (*Gpx4* ^Δhepa^) in hepatocytes compared with *Gpx4* ^*f*/f^ animals (referred to as wild type [WT]) (Figure 2A). Consistent with this, histopathological examination showed that *Gpx4* ^Δhepa^ animals developed a more severe phenotype with multiple infarcts and areas of necrosis (Figure 2B).

### 3.2 Loss of *Gpx4* in Hepatocytes Enhances Susceptibility to Apoptotic Cell Death

Next, we determined which cell death mechanism was responsible for acute liver injury. Given that the deletion of Gpx4 increases susceptibility to ferroptosis, we initially sought to ascertain the presence of products of lipid peroxidation. 4‐Hydroxynonenal (4HNE) is a reactive toxic aldehyde that forms adducts with membrane proteins and is a known marker of lipid peroxidation in ferroptosis. No difference was observed in the number of 4HNE‐positive areas between the two CCl_4_‐treated groups (Figure 2C). Additionally, no changes were observed in malondialdehyde (MDA) or acyl‐CoA synthetase long chain family member 4 (ACSL4) levels between the untreated and treated mice or between genotypes (Figure S2A–C), indicating that cell death is independent of ferroptosis. Subsequently, we performed TUNEL staining and detected a strong and significantly more positive cells in *Gpx4* ^Δhepa^ compared to WT livers (Figure 2D), suggesting that apoptotic cell death might be involved. This result was strengthened by cleaved caspase‐3 staining demonstrating a significant increase of positive cells in *Gpx4* ^Δhepa^ livers compared to WT controls (Figure 2D). Additionally, we observed an increase in the expression of pro‐apoptotic mediators (Figure 2E) and mitochondrial biogenesis markers (Figure S2D).

Moreover, the formation of apoptotic bodies leads to the release of damage‐associated molecular patterns, which subsequently activate resident liver macrophages, the Kupffer cells, thereby initiating an immune response. Consistent with this, flow cytometry analysis of liver‐infiltrating immune populations showed an increased number of macrophages (Figure 2F), while the numbers of other immune populations, such as neutrophils and lymphocytes, remained unchanged (data not shown). The increased macrophage infiltration in *Gpx4* ^Δhepa^ livers was validated by quantifying the number of cells co‐expressing Cd11b and F4/80, which showed a significant increase compared to the WT group (Figure S3A). In addition, we observed a significant decrease in the mRNA expression of anti‐inflammatory M2 markers in *Gpx4* ^Δhepa^ livers, although no changes in M1 markers were detected (Figure S3B).

Since GPX4 has been identified in several RCDs, we assessed necroptosis and pyroptosis effector protein levels by immunodetection. However, no significant differences were observed between the CCl_4_‐treated groups (Figure S3C).

To confirm that apoptosis underlies cell death in this model, a 24‐h experiment was performed to capture early events without bias from surviving cells. Gene set enrichment analysis showed that apoptotic mRNA machinery was active in WT mice after CCl_4_ administration (Figure S3D). However, *Gpx4*‐deficient hepatocytes exhibited higher levels of cleaved caspase‐3, a key effector of apoptosis, and other apoptotic markers, consistent with the increased damage observed in the biochemical analyses. These findings suggest that *Gpx4* ^Δhepa^ mice are more susceptible to apoptosis, leading to more severe cell death resulting in faster and more severe cell death.

### 3.3 *Gpx4* ^Δhepa^ Mice Show Increased Apoptosis in an Acute Model of Cholestasis

**FIGURE 3:**
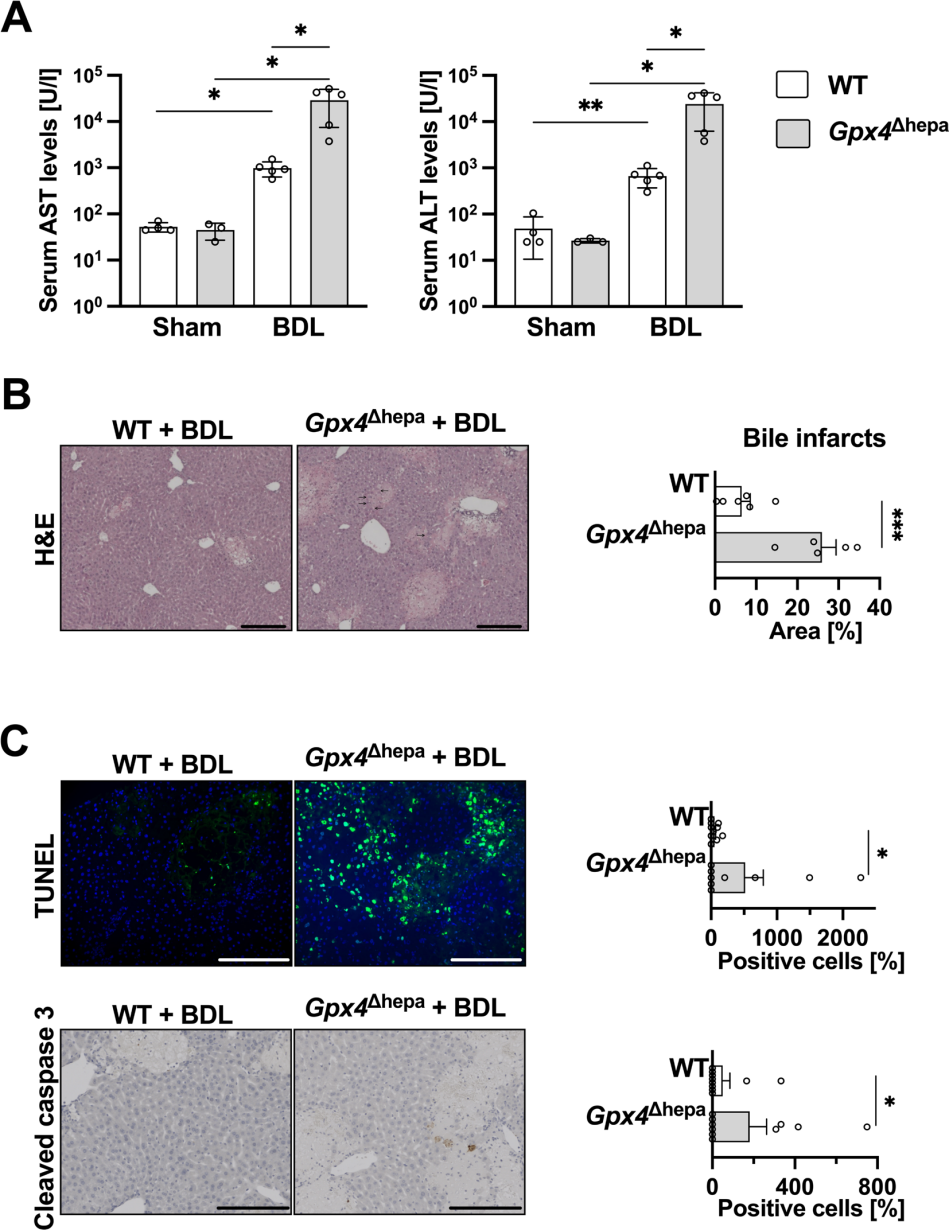
Liver injury and apoptosis are increased in *Gpx4* ^Δhepa^ mice after BDL. (A) Serum transaminases levels (left: AST, right: ALT) in sham and BDL WT (sham, *n* = 4; BDL, *n* = 5; white) and *Gpx4* ^Δhepa^ (sham, *n* = 3, BDL, *n* = 5; light grey) mice. (B) Left: H&E stainings of liver sections from WT and *Gpx4* ^Δhepa^ mice 48 h after BDL. Scale bars = 100 μm. Arrows indicate cells undergoing apoptosis. Right: Necrosis area ratios comparison among WT and *Gpx4* ^Δhepa^ mice. (C) Above: TUNEL staining. Left: Representative images. Scale bar = 200 μm. Right: Percentage of positive cells stained for TUNEL per view field relative to the WT group. Bottom: Cleaved caspase 3 IHC staining. Left: Representative images. Scale bar = 200 μm. Right: Percentage of positive cells stained for cleaved caspase 3 per view field relative to the WT group. Data are expressed as ± SEM from BDL WT (*n* = 5) and *Gpx4* ^Δhepa^ (*n* = 5). Ordinary one‐way analysis of variance (ANOVA) with Tukey's multiple comparison test was performed for panel A; unpaired t test comparing *Gpx4* ^Δhepa^ to WT mice was conducted for panel (B–C). *p < 0.05; **p < 0.01; ***p < 0.001. Aspartate transaminase, AST; alanine transaminase, ALT; bile duct ligation, BDL; haematoxylin–eosin, H&E.

Since we found increased apoptotic cell death but no significant evidence of ferroptotic cell death in *Gpx4* ^Δhepa^ livers after CCl_4_ treatment, we expanded our analysis to further investigate this unexpected finding by including a second model and performing an acute cholestasis‐induced ALF model. Forty‐eight hours after BDL, WT animals exhibited severely impaired liver function, as revealed by exponentially elevated serum transaminase levels compared to the sham treated group. However, *Gpx4* ^Δhepa^ animals showed significantly exacerbated liver damage compared to the WT group, as evidenced by a 29‐ and 36‐fold increase in AST and ALT levels, respectively (Figure 3A). The results of H&E staining demonstrated the existence of extensive necrotic areas within the *Gpx4* ^Δhepa^ livers, which exhibited cells with distinctive nuclear characteristics, including pyknosis and annular chromatin condensation. These observations, in line with the hallmarks of apoptosis, were markedly more prevalent and extensive compared to the findings observed in the WT mice (Figure 3B). Consistent with the observed mechanism responsible for cell death in the CCl_4_ model, we detected a significant increase in TUNEL and cleaved caspase‐3 positive cells after BDL in *Gpx4* ^Δhepa^ livers (Figure 3C). These results suggest that *Gpx4* in hepatocytes is crucial in determining their susceptibility toward apoptosis‐mediated cell death after acute liver injury.

### 3.4 *Keap1* Co‐Deletion in *Gpx4*‐Deficient Hepatocytes Induces the Antioxidant Response and Protects Against Damage

Thus far, our results suggest that the absence of *Gpx4* in hepatocytes exacerbates the degree of liver injury. Since GPX4 is a phase II antioxidant enzyme, we hypothesised that the induction of other NRF2‐dependent phase II enzymes could compensate for the absence of *Gpx4*. Hence, we generated mice with a combined deletion of *Gpx4* and *Keap1* in hepatocytes (*Gpx4* ^Δhepa^ *Keap1* ^Δhepa^ mice).

**FIGURE 4:**
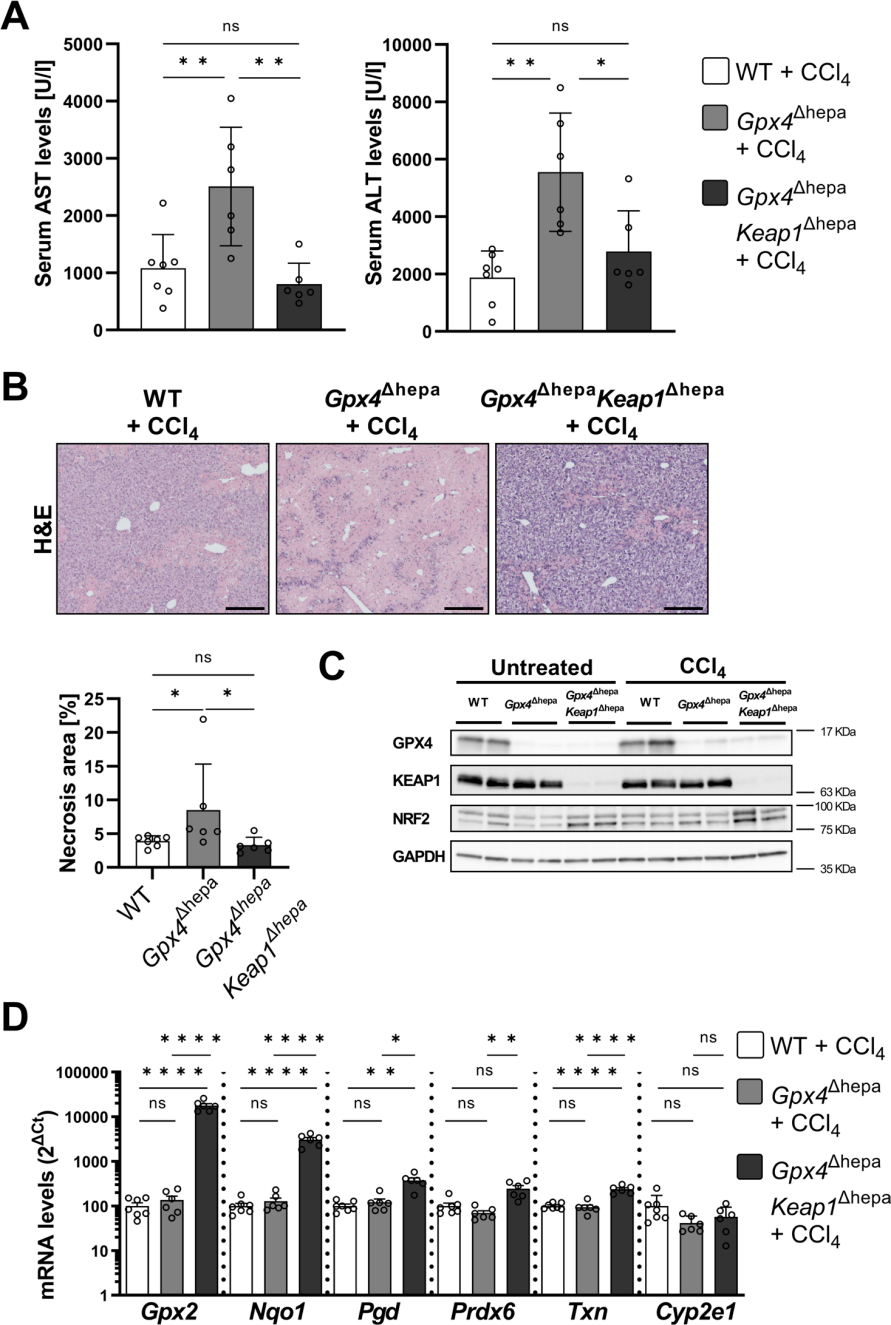
*Keap1* co‐deletion in *Gpx4*‐deficient hepatocytes rescues liver injury after CCl_4_ treatment. (A) Serum transaminases levels (left: AST, right: ALT) in CCl_4_‐treated WT (*n* = 7; white), *Gpx4* ^Δhepa^ (*n* = 6; grey), and *Gpx4* ^Δhepa^ *Keap1* ^Δhepa^ (*n* = 6; charcoal) mice. (B) Above: H&E stainings of liver sections from WT and *Gpx4* ^Δhepa^, and *Gpx4* ^Δhepa^ *Keap1* ^Δhepa^ mice 48 h after CCl_4_ treatment. Scale bars = 100 μm. Bottom: Necrosis area ratios comparison among groups. (C) Immunoblot analysis of liver extracts. GAPDH control shows homogeneous loading of the protein in each line. (D) level mRNA levels of phase I (*Cyp2e1*) and II (*Gpx2, Nqo1, Pgd, Prdx6*, and *Txn*) antioxidant enzymes. Data are expressed as ± SEM from CCl_4_‐treated WT (*n* = 7; white), *Gpx4* ^Δhepa^ (*n* = 6; grey), and *Gpx4* ^Δhepa^ *Keap1* ^Δhepa^ (*n* = 6; charcoal) mice. Ordinary one‐way analysis of variance (ANOVA) with Tukey's multiple comparison test was performed. ns, not significant; *p < 0.05; **p < 0.01; ****p < 0.0001. Aspartate transaminase, AST; alanine transaminase, ALT; haematoxylin–eosin, H&E; glutathione peroxidase 2, Gpx2; NAD(P)H:Quinone dehydrogenase 1, Nqo1; phosphogluconate dehydrogenase, Pgd; peroxiredoxin 6, Prdx6; thioredoxin, Txn; cytochrome P450 family 2 subfamily E member 1, Cyp2e1.

CCl_4_ treatment of *Gpx4* ^Δhepa^ *Keap1* ^Δhepa^ mice induced liver injury as evidenced by increased transaminase levels compared to untreated controls (see in Figure S4). However, this increase was significantly attenuated compared to *Gpx4* ^Δhepa^ mice and on the same level as found in WT animals (Figure 4A). These results were further confirmed by our histopathological analysis by performing H&E stainings, where *Gpx4* ^Δhepa^ *Keap1* ^Δhepa^ animals rescued the size of necrotic areas to a level as found in WT livers after CCl_4_ challenge (Figure 4B).

To validate our findings, we next confirmed that *Keap1* deletion induces a NRF2‐dependent antioxidant response. Double deletion of *Gpx4* and *Keap1* in hepatocytes resulted in increased NRF2 protein levels, which were further elevated after CCl_4_‐induced injury (Figure 4C). This was accompanied by increased expression of phase II antioxidant enzymes, including *Gpx2*, *Nqo1*, *Pgd*, *Prdx6*, and *Txn* (Figure 4D). In contrast, the expression of the phase I enzyme cytochrome P450 family 2 subfamily E member 1 (*Cyp2e1*), which is responsible for CCl_4_ metabolism, remained unchanged (Figure 4D). In addition, enzymes belonging to the glutathione peroxidase‐active family (*Gstas*), which metabolise various toxins and products of oxidative stress, were upregulated in *Gpx4* ^Δhepa^ *Keap1* ^Δhepa^ mice independent of CCl_4_ administration (Figure S4B).

Furthermore, NRF2 regulates enzymes involved in glutathione (GSH) biosynthesis. In the *Gpx4* ^Δhepa^ *Keap1* ^Δhepa^ group, we observed upregulation of enzymes involved in *de novo* GSH synthesis, such as glutamate‐cysteine ligase catalytic subunit (*Gclc*), and enzymes responsible for converting oxidised glutathione (GSSG) to its reduced form, such as glutathione‐disulfide reductase (*Gsr*) (Figure S4B). To evaluate whether these changes affected glutathione availability and the antioxidant response induced by NRF2 activation, we measured GSH and GSSG levels. While CCl_4_ administration increased oxidised glutathione levels in WT and *Gpx4* ^Δhepa^ groups (Figure S4C), the *Gpx4* ^Δhepa^ *Keap1* ^Δhepa^ group exhibited a GSH/GSSG ratio indicative of a shift toward a more favourable antioxidant balance, albeit without clear statistical significance (Figure S4D).

### 3.5 *Keap1* Deletion Has an Anti‐Apoptotic Effect

**FIGURE 5:**
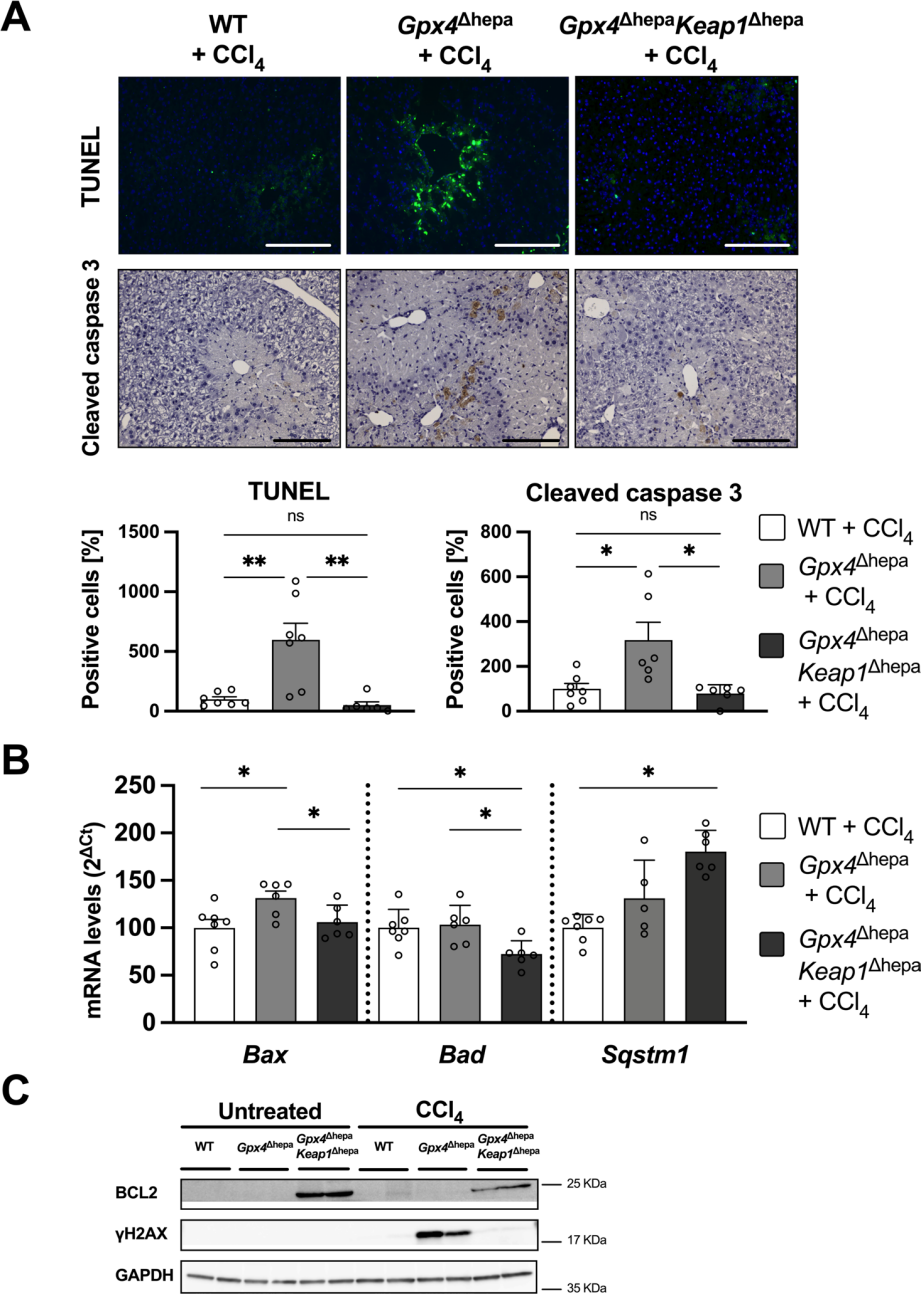
Apoptosis is inhibited in *Gpx4* ^Δhepa^ *Keap1* ^Δhepa^. (A) Above: Representative images for TUNEL staining and cleaved caspase 3 IHC. Scale bar = 200 μm. Bottom: Percentage of positive cells stained for TUNEL (left) and cleaved caspase 3 (right) per view field relative to the WT group. (B) mRNA levels for pro‐apoptotic markers (*Bax* and *Bad*) and mitophagy‐related gene *Sqstm1*. (C) Immunoblot analysis of liver extracts. GAPDH control shows equal loading of the protein in each line. Data are expressed as ± SEM from CCl_4_‐treated WT (*n* = 7; white), *Gpx4* ^Δhepa^ (*n* = 6; grey), and *Gpx4* ^Δhepa^ *Keap1* ^Δhepa^ (*n* = 6; charcoal) mice. Ordinary one‐way analysis of variance (ANOVA) with Tukey's multiple comparison test was performed. ns, not significant; *p < 0.05. BCL2 associated X apoptosis regulator, Bax; BCL2 associated agonist of cell death, Bad; BCL2 apoptosis regulator, BCL2; gamma H2A.X variant histone, γH2AX; sequestesome‐1, Sqstm1.

Our findings identified apoptosis as the primary mechanism of cell death following CCl_4_‐induced liver injury, with this process being enhanced in *Gpx4*‐depleted hepatocytes. To further investigate apoptosis in *Gpx4* ^Δhepa^ *Keap1* ^Δhepa^ mice, we performed TUNEL assay and cleaved caspase‐3 staining. We observed a marked reduction in the number of hepatocytes undergoing apoptosis compared to *Gpx4* ^Δhepa^ livers (Figure 5A). Additionally, the expression levels of the pro‐apoptotic genes *Bad* and *Bax* were lower, while sequestosome‐1 (*Sqstm1*, also known as *p62*) expression was higher in the *Gpx4* ^Δhepa^ *Keap1* ^Δhepa^ group compared to the *Gpx4* ^Δhepa^ group (Figure 5B).

Notably, an increase in the anti‐apoptotic protein BCL2 was observed in *Keap1* deficient mice, both at baseline and after CCl_4_ administration (Figure 5C). This finding is consistent with the reduction in apoptosis observed in the *Gpx4* ^Δhepa^ *Keap1* ^Δhepa^ group. In addition, since the TUNEL assay detects DNA fragmentation typical of apoptotic cells, we evaluated the levels of γH2AX and observed that the administration of CCl_4_ leads to enhanced levels of γH2AX in the *Gpx4* ^Δhepa^ group, which were rescued in *Gpx4* ^Δhepa^ *Keap1* ^Δhepa^ livers (Figure 5C).

## 4 Discussion

Acute liver injury can progress to liver failure, a life‐threatening event with few therapeutic options other than liver transplantation. In patients with ALI, early administration of N‐acetylcysteine has been widely used and in early stages might be helpful for all ALF patients as it has an anti‐oxidative effect. However, it is especially effective and specific in cases associated with acetaminophen (APAP) intoxication. Hence, up to date, there is a lack of evidence for positive outcomes in non‐APAP cases, especially in late stages [1]. In Germany, prospective studies place APAP intoxication as the third cause of ALI, after non‐APAP intoxication and viral etiologies [21, 22]. For non‐APAP patients who progress to ALF, the only curative option is liver transplantation [21].

For drug‐induced liver injury (DILI) it has been postulated that intercellular stress in hepatocytes is an initiating factor essential to trigger liver injury [23]. Here, GPX4 appears to be particularly crucial in acute situations, as evidenced by our observation that GPX4 levels are reduced in patients with ALF compared to controls or patients with ACLF (Figure 1A). To verify these findings, we used CCl_4_ to induce ALI in mice. CCl_4_ is metabolised by CYP2E1, and reactive free radicals initiate peroxidation of lipids, especially of polyunsaturated fatty acids, leading to membrane disruption, resulting in cell damage and liver dysfunction. Thus, in this context, the antioxidant function of GPX4 in maintaining lipid homeostasis seems to be of vital importance. Indeed, after CCl_4_ treatment, *Gpx4* is the only phase II antioxidant enzyme that is altered (Figure 1B–D). Notably, *Prdx6*, which shares substrate affinity for peroxidised phospholipids with GPX4 and could be beneficial after CCl_4_ injury, is not modified (Figure 1D). These data support the previous notion that the role of GPX4 in redox homeostasis is more critical than the activity of PRDX6 [24].

To functionally investigate the role of GPX4, we generated mice with deletion of *Gpx4* in hepatocytes. The critical role of GPX4 has been known since the development of *Gpx4* global knockout animals, which results in embryonic lethality. Carlson developed a conditional *Gpx4* knockout in hepatocytes, and although the mice were viable, they died in the first days of life from extensive liver degeneration. However, by feeding them a diet enriched in vitamin E, they were able to reverse this genotype‐related damage [11]. By adapting these recommendations, we succeeded in breeding *Gpx4* ^Δhepa^ mice without observing any genotype‐related burden. In Carlson's work, *Gpx4* ^Δhepa^ mice exhibited genotype‐associated negative effects up to 4 weeks after withdrawal from a vitamin E‐rich diet [11]. In our case, after 4 weeks on a standard diet, we found no differences in the health status between Gpx4‐deficient and Gpx4‐efficient littermates. Consequently, we proceeded to perform ALI induction experiments.

Induction of ALI with CCl_4_ caused severe impairment of liver function in both *Gpx4* ^*f*/f^ and *Gpx4* ^Δhepa^ animals, as observed by serum transaminase levels (Figure 2A). The absence of *Gpx4* significantly exacerbated the injury, as indicated by liver markers (Figure 2A) and histologic analysis (Figure 2B). Along with the tissue damage, we observed an increase in macrophages, which was confirmed by flow cytometry (Figure 2F) and immunohistochemistry (Figure S3A). Notably, upon characterising the macrophage phenotype as pro‐inflammatory M1 or anti‐inflammatory M2, only a reduction in M2 markers was observed, whereas no variation was evident in the expression levels of M1 markers (Figure S3B). This discrepancy may be attributed to the fact that the peak expression of M1 markers occurs earlier (at 8 h post‐injury) in comparison to that of the M2 markers [25]. Consequently, the observed reduction in RNA levels of *Mrc1*, *Cd163*, and *Il10* suggests a reduced anti‐inflammatory polarisation of macrophages in *Gpx4* ^Δhepa^ livers.

We then proceeded to identify the cell death mechanism responsible for hepatocyte death. Surprisingly, we observed no evidence of lipid peroxidation in *Gpx4* ^Δhepa^ mice (Figure 2C and Figure S2A–C) and, therefore, had to exclude the involvement of ferroptosis in this model. The absence of GPX4 is mainly associated with increased susceptibility to ferroptosis, but it has also been described to be associated with apoptosis, pyroptosis, and necroptosis [6]. We did not detect changes in the levels of necrosomal complex proteins (receptor‐interacting serine/threonine kinase 3 [RIPK3] and mixed lineage kinase domain like pseudokinase [MLKL]), which are responsible for necroptosis, or the pyroptosis executioner protein, gasdermin D (GSDMD) (Figure S3C). However, by investigating markers of apoptosis, we showed a clear involvement of this RCD in the damage observed in the *Gpx4* ^Δhepa^ livers (Figure 2D,E).

The findings were confirmed by RNA‐Seq analysis performed on a group of mice 24 h after CCl_4_ administration, which identified several GO terms positively associated with apoptosis among the top enriched pathways. The presence of the terms “positive regulation of TNF production” and “cellular response to TNF” suggests activation of the extrinsic pathway of apoptosis in *Gpx4* ^*f*/f^ mice compared to the control group (Figure S3D). The identification of seemingly opposing terms, such as “negative regulation of TNF production” and “negative regulation of cell death”, likely reflects feedback mechanism rather than apoptosis suppression, indicating a dynamic and continuously evolving process. Integrating these results with our earlier data from the 48‐h CCl_4_ model, we conclude that apoptosis is the predominant mechanism responsible for liver damage in this hepatotoxicity model.

Furthermore, the absence of *Gpx4* has been shown to exacerbate pre‐existing apoptosis, a phenomenon that may be mediated by elevated levels of *Bax*, which serves as a link between the extrinsic and intrinsic pathways of apoptosis, thereby amplifying the apoptotic response [26]. The observed increase in mitochondrial biogenesis in the *Gpx4* ^Δhepa^ group (Figure S2D) provides further evidence of the involvement of intrinsic apoptosis, namely mitochondrial apoptosis. This observation aligns with its established role as a hallmark of late‐stage apoptotic cells [27].

To confirm that apoptosis is responsible for the death of *Gpx4*‐deficient hepatocytes in acute injury situations, we implemented a second ALI model triggering acute cholestasis. The liver damage induced by BDL is dramatically higher in the *Gpx4* ^Δhepa^ group, as evidenced by transaminase levels (Figure 3A). Histologic analysis, in conjunction with TUNEL assays and cleaved caspase‐3 staining (Figure 3B,C), is indicative of cells undergoing apoptosis. However, it is pertinent to acknowledge that BDL‐derived cell death in mice has been extensively documented to exhibit predominantly necrosis‐like characteristics [28].

Despite the existence of conflicting opinions regarding the role of ferroptosis in hepatotoxicity, there is currently no clear consensus on this matter. The precise relationship between LPO and the mechanism of DILI remains uncertain, with the possibility that it may either result from, or be a contributing factor to, the damage itself [23]. Some studies have indicated that in APAP models, if there is a normal basal functioning of antioxidant defences, LPO does not contribute significantly to the injury induced by liver toxicity [29]. However, in cases of other hepatotoxic drugs, there appears to be a correlation between increased LPO and damage [30]. Further research is necessary in this type of model to better define the role of LPO in the mechanism of DILI. In addition to the liver, the kidney is an important organ in drug metabolism and can suffer from acute kidney injury caused by drugs. There is growing evidence that ferroptosis is involved in drug‐induced kidney damage, as observed with folic acid [31, 32] cisplatin [33, 34]. It is noteworthy that in cases of cisplatin‐induced acute kidney injury, numerous cell death mechanisms are involved, including apoptosis, necroptosis, and ferroptosis [35]. Oxidative stress plays a crucial role. Indeed, boosting of antioxidant defences has been employed as a means of combating injury induced by cisplatin [36, 37].

The deletion of *Keap1* in parallel with *Gpx4* in hepatocytes resulted in a complete reversal of CCl_4_‐induced liver damage, making it comparable to WT controls (Figure 4A,B). KEAP1 targets NRF2 for constant degradation; therefore, its deletion leads to NRF2 accumulation (Figure 4C) and the upregulation of NRF2‐dependent second‐line antioxidant enzymes (Figure 4C and Figure S4B). Given the extensive regulatory scope of NRF2, which includes over 250 genes [38], the protective mechanism is likely multifactorial. Among these, the upregulation of *Gsta* family members probably contributes to the clearance of oxidative stress byproducts, thereby reducing ROS propagation (Figure S4B). Furthermore, an increased expression of enzymes involved in glutathione biosynthesis was observed (Figure S4B), which correlated with a trend toward a higher GSH/GSSG ratio in the *Gpx4* ^Δhepa^ *Keap1* ^Δhepa^ group, indicative of an improved antioxidant defence (Figure S4D). Another potential contributor to the protective mechanism involves the upregulation of the mitophagy‐related gene *Sqstm1* (Figure 5B) whose transcription is NRF2‐dependent. In the *Gpx4* ^Δhepa^ group, CCl_4_ administration amplified apoptotic signalling; however, increased mitophagy in the *Gpx4* ^Δhepa^ *Keap1* ^Δhepa^ group may act as a compensatory response, mitigating mitochondrial contributions to apoptosis. This hypothesis is further substantiated by the observation of diminished mRNA levels of the pro‐apoptotic genes *Bad* and *Bax* in the *Gpx4* ^Δhepa^ *Keap1* ^Δhepa^ group (Figure 5B). The observed reduction in DNA damage, as indicated by decreased γH2AX levels, further supports the protective mechanisms that mitigate apoptosis (Figure 5C).

Notably, the ablation of *Keap1* has consequences beyond the activation of Nrf2. KEAP1 interacts with numerous proteins and exerts a significant influence on cell function [39]. The protein BCL2 is a substrate of KEAP1 [40]. Our previous research demonstrated that mice with a deletion of *Keap1* exhibit impaired degradation of BCL2, leading to its accumulation [41]. Given the anti‐apoptotic effect of BCL2, it can be postulated that the observed increase in the *Gpx4* ^Δhepa^ *Keap1* ^Δhepa^ group may contribute to rescuing apoptosis in our model (Figure 5C). Due to the extensive range of interactions of both Nrf2 and KEAP1, it is challenging to identify which is the key factor responsible for the reversal of liver damage in our model. Therefore, it is possible that joint effects contribute to the reversal of the phenotype found in *Gpx4* ^Δhepa^ *Keap1* ^Δhepa^ animals.

Our results suggest that stimulating NRF2‐dependent endogenous antioxidant defences may be a potential treatment for ALI not associated with APAP intoxication. Because APAP overdose is the leading cause of ALI in countries with greater access to OTC drugs, such as the United States or the United Kingdom [1, 42, 43], there are more studies related to this aetiology. Therefore, due to the smaller number of studies on cases of ALI not related to acetaminophen and the lack of therapeutic options, there is an urgent need to study this pathological situation to find new therapeutic approaches. Among NRF2 agonists, there are numerous compounds of natural origin [44]. While the majority of these have only been tested in experimental models [45], there are a few exceptions, such as bicyclol, which has been approved by the Chinese Food and Drug Administration and has been shown to have a hepatoprotective effect in DILI [46, 47]. Additionally, to avoid off‐target effects, there are several studies investigating the use of nano‐encapsulation of NRF2 activators [48], which may increase the likelihood of translation to the clinic. A Phase I clinical trial (NCT03902002) is currently underway to evaluate the use of omaveloxolone [49], a drug approved by the FDA in 2023 for the treatment of Friedreich's ataxia, in patients with severe hepatic impairment [50].

In conclusion, we demonstrate that *Gpx4* plays a crucial role in non‐APAP cases of ALI. Lack of expression in hepatocytes leads to increased apoptosis‐mediated cell death. Importantly, in vivo, inducing a NRF2‐dependent antioxidant response in hepatocytes triggers a protective effect. Therefore, we provide new evidence that targeting *Keap1* and activating NRF2 is a promising therapeutic intervention in the management of patients with ALI not related to acetaminophen overdosing.

## Author Contributions

Conceptualization: L.C., J.G., and C.T. Methodology: L.C., J.G., M.K., J.H., and C.T. Investigation: L.C., J.G., C.W., J.D., M.K., J.R., and K.E. Formal analysis: L.C., J.G., C.W., J.D., M.K., J.R., K.E., and N.G. Resources: D.J., J.H., and C.T. Data curation: L.C. and J.G. Visualisation: L.C. and J.G. Writing – original draft: L.C. and C.T. Writing – review and editing: all authors. Supervision: L.C. and C.T. Project administration: C.T. Funding acquisition: L.C. and C.T.

## Ethics Statement

This retrospective study was carried out according to the guidelines of the Declaration of Helsinki and approved by the local ethics committee of the Medical Faculty of RWTH Aachen University (EK23‐350). Patient consent was waived due to working with diagnostic material approved by the local ethics committee of the Medical Faculty of RWTH Aachen University (EK23‐350). All animal experiments were performed under approvals from the appropriate authorities for animal welfare (LANUV file ref. AZ‐84‐02.04.2019.A490, AZ‐84‐02.04.2016.A080 and AZ‐81‐02.04.2020.A390) and in compliance with EU Directive 2010/63/EU.

## Conflicts of Interest

The authors declare no conflicts of interest.

## Supporting information


**Figure S1.** Confirmation of GPX4 deletion in hepatocytes. (A) GPX4 IHC of WT (left) and *Gpx4* ^Δhepa^ (right) mice. Scale bar = 100 μm. (B) Immunoblot analysis of liver extracts. GAPDH control shows equal loading of the protein in each line.

**Figure S2.** Cell death mechanisms evaluation after CCl_4_ treatment. (A) MDA quantification in untreated and CCl_4_‐treated WT (untreated, *n* = 4; treated, *n* = 7; white) and *Gpx4* ^Δhepa^ (untreated, *n* = 3, treated, *n* = 6; grey) mice. (B) RT‐qPCR of *Acsl4* in untreated and CCl_4_‐treated WT (untreated, *n* = 4; treated, *n* = 7; white) and *Gpx4* ^Δhepa^ (untreated, *n* = 3, treated, *n* = 6; grey) mice. (C) Left: immunoblot analysis of ACSL4. GAPDH control shows equal loading of the protein. Right: quantification of protein levels, normalised by GAPDH. D RT‐qPCR of mitochondrial biogenesis related genes *Cytb* and *Mt‐nd1*. Data are expressed as ± SEM; ns, not significant; * *p* < 0.05; ordinary one‐way ANOVA with Tukey's multiple comparison test was performed for panels A–C, unpaired *t* test comparing *Gpx4* ^Δhepa^ to WT mice was conducted for panel D. Acyl‐CoA synthetase long chain family member 4, ACSL4; malondialdehyde, MDA; mitochondrially encoded cytochrome b, Cytb; mitochondrially encoded NADH:ubiquinone oxidoreductase core subunit 1, Mt‐nd.

**Figure S3.** Analysis of immune infiltration, cell death pathways and GSEA in CCl_4_‐treated mice. (A) Immunofluorescence staining of CD11b^+^F4/80^+^ macrophages in liver. Left: representative merged images with CD11b (magenta), F4/80 (green) and DAPI (blue) channels are shown. Scale bar = 200 μm. Right: quantification of cells co‐expressing CD11b and F4/80. (B) mRNA levels of macrophage polarisation markers (M1: *Il6, Tnfα, Il1b*; M2: *Mrc1, Cd163, Il10*) in WT and *Gpx4* ^Δhepa^ treated with CCl_4_. Data are expressed as ± SEM from CCl_4_‐treated WT (*n* = 7) and *Gpx4* ^Δhepa^ (*n* = 6). (C) Left: Immunoblot analysis of liver extracts for proteins involved in necroptosis (pRIPK3, RIPK3, and pMLKL) and pyroptosis (GSDMD). GAPDH control shows equal loading of the protein. Right: quantification of protein levels, normalised as indicated. D GO enrichment analysis of DEGs related to cell death in WT untreated versus WT + 24 h CCl_4_ mice. Data are expressed as ± SEM; ns, not significant; ** *p* < 0.01; unpaired t test comparing *Gpx4* ^Δhepa^ to WT mice was conducted for panel A–C. CD163 molecule, Cd163; F4/80 glycoprotein, F4/80; gasdermin D, GSDMD; interleukin 1b, Il1b; interleukin 6, Il6; interleukin 10, Il10; integrin alpha M, CD11b; mannose receptor C‐type 1, Mrc1; phospho mixed lineage kinase domain like pseudokinase, pMLKL; (phospho‐) receptor‐interacting serine/threonine kinase 3, (p‐) RIPK3; tumour necrosis factor alpha, Tnfα.

**Figure S4.** Transaminases levels of untreated and CCl_4_‐treated *Gpx4* ^Δhepa^ *Keap1* ^Δhepa^ mice. (A) AST (left) and ALT (right) levels in serum from untreated (*n* = 4) and CCl_4_‐treated (*n* = 6) mice. (B) RT‐qPCR analysis for glutathione peroxidase‐active family members 1, 2 and 4 (above) and glutathione biosynthesis related genes (*Gclc* and *Gsr*) (below) from CCl_4_‐treated WT (*n* = 7; white), *Gpx4* ^Δhepa^ (*n* = 6; grey), and *Gpx4* ^Δhepa^ *Keap1* ^Δhepa^ (*n* = 6; charcoal) mice. (C) GSSG quantification in CCl_4_‐treated versus untreated WT (untreated *n* = 5, CCl_4_ *n* = 3; white), *Gpx4* ^Δhepa^ (untreated *n* = 5, CCl_4_ *n* = 3; grey), and *Gpx4* ^Δhepa^ *Keap1* ^Δhepa^ (untreated *n* = 5, CCl_4_ *n* = 5; charcoal) mice. D GSH/GSSG ratio in CCl_4_‐treated mice. Data are expressed as ± SEM; ns, not significant; * *p* < 0.05; ** *p* < 0.01; unpaired t test comparing treated to untreated *Gpx4* ^Δhepa^ *Keap1* ^Δhepa^ mice was conducted for panels A and C; for panels B and D ordinary one‐way analysis of variance (ANOVA) with Tukey's multiple comparison test was performed. Aspartate transaminase, AST; alanine transaminase, ALT; glutathione peroxidase‐active, Gsta; glutamate‐cysteine ligase catalytic subunit, Gclc; glutathione‐disulfide reductase, Gsr, oxidised form of glutathione, GSSG; reduced form of glutathione, GSH.

**Table S1.** Etiologies associated with patients with liver failure.

**Table S2.** Antibody list.

**Table S3.** Primer list for RT‐qPCR.

## Data Availability

The data that support the findings of this study are available from the corresponding author upon reasonable request.

## References

[1] D. G. Koch , J. L. Speiser , V. Durkalski , et al., “The Natural History of Severe Acute Liver Injury,” American Journal of Gastroenterology 112, no. 9 (2017): 1389–1396, 10.1038/ajg.2017.98. 28440304 PMC5587371

[2] H. Jaeschke and A. Ramachandran , “Reactive Oxygen Species in the Normal and Acutely Injured Liver,” Journal of Hepatology 55, no. 1 (2011): 227–228, 10.1016/j.jhep.2011.01.006. 21238521 PMC3117914

[3] V. Unsal , M. Cicek , and İ. Sabancilar , “Toxicity of Carbon Tetrachloride, Free Radicals and Role of Antioxidants,” Reviews on Environmental Health 36, no. 2 (2021): 279–295, 10.1515/reveh-2020-0048. 32970608

[4] C. Tonelli , I. I. C. Chio , and D. A. Tuveson , “Transcriptional Regulation by Nrf2,” Antioxidants & Redox Signaling 29, no. 17 (2018): 1727–1745, 10.1089/ars.2017.7342. 28899199 PMC6208165

[5] Q. Ma , “Role of Nrf2 in Oxidative Stress and Toxicity,” Annual Review of Pharmacology and Toxicology 53, no. 1 (2013): 401–426, 10.1146/annurev-pharmtox-011112-140320. PMC4680839 23294312

[6] Y. Xie , R. Kang , D. J. Klionsky , and D. Tang , “GPX4 in Cell Death, Autophagy, and Disease,” Autophagy 19, no. 10 (2023): 2621–2638, 10.1080/15548627.2023.2218764. 37272058 PMC10472888

[7] H. Ye , L. J. Nelson , M. G. del Moral , E. Martínez‐Naves , and F. J. Cubero , “Dissecting the Molecular Pathophysiology of Drug‐Induced Liver Injury,” World Journal of Gastroenterology 24, no. 13 (2018): 1373–1385, 10.3748/WJG.V24.I13.1373. 29632419 PMC5889818

[8] Y. Zheng , B. Cui , W. Sun , et al., “Potential Crosstalk Between Liver and Extra‐Liver Organs in Mouse Models of Acute Liver Injury,” International Journal of Biological Sciences 2020, no. 7 (2020): 1166–1179, 10.7150/ijbs.41293. PMC7053327 32174792

[9] W. S. Yang , R. Sriramaratnam , M. E. Welsch , et al., “Regulation of Ferroptotic Cancer Cell Death by GPX4,” Cell 156, no. 1–2 (2014): 317–331, 10.1016/j.cell.2013.12.010. 24439385 PMC4076414

[10] C. Kellendonk , C. Opherk , K. Anlag , G. Nther Schü , and T. F. Ois , “Hepatocyte‐Specific Expression of Cre Recombinase,” Genesis (New York, N.Y.: 2000) 26 (2000): 151, 10.1002/(SICI)1526-968X(200002)26:2. 10686615

[11] B. A. Carlson , R. Tobe , E. Yefremova , et al., “Glutathione Peroxidase 4 and Vitamin E Cooperatively Prevent Hepatocellular Degeneration,” Redox Biology 9 (2016): 22–31, 10.1016/j.redox.2016.05.003. 27262435 PMC4900515

[12] D. Scholten , J. Trebicka , C. Liedtke , and R. Weiskirchen , “The Carbon Tetrachloride Model in Mice,” Laboratory Animals 49 (2015): 4–11, 10.1177/0023677215571192. 25835733

[13] C. G. Tag , S. Sauer‐Lehnen , S. Weiskirchen , et al., “Bile Duct Ligation in Mice: Induction of Inflammatory Liver Injury and Fibrosis by Obstructive Cholestasis,” JoVE (Journal of Visualized Experiments) 96, no. 96 (2015): e52438, 10.3791/52438. PMC4354634 25741630

[14] R. Aparecido , B. Nucci , W. Jacob‐Filho , et al., “Color Deconvolution as a Simple and Rapid Tool in Quantitative Biomedical Research,” Journal of Health and Allied Sciences NU 14 (2024): 146–148, 10.1055/s-0043-1768067.

[15] R. Hassan , Z. Hobloss , M. Myllys , et al., “Acetaminophen Overdose Causes a Breach of the Blood‐Bile Barrier in Mice but not in Rats,” Archives of Toxicology 98 (2024): 1533–1542. 38466352 10.1007/s00204-024-03705-6

[16] B. MacLean , D. M. Tomazela , N. Shulman , et al., “Skyline: An Open Source Document Editor for Creating and Analyzing Targeted Proteomics Experiments,” Bioinformatics 26, no. 7 (2010): 966–968, 10.1093/bioinformatics/btq054. 20147306 PMC2844992

[17] R. Patro , G. Duggal , M. I. Love , R. A. Irizarry , and C. Kingsford , “Salmon Provides Fast and Bias‐Aware Quantification of Transcript Expression,” Nature Methods 14, no. 4 (2017): 417–419, 10.1038/NMETH.4197. 28263959 PMC5600148

[18] M. I. Love , C. Soneson , P. F. Hickey , et al., “Tximeta: Reference Sequence Checksums for Provenance Identification in RNA‐Seq,” PLoS Computational Biology 16, no. 2 (2020): e1007664, 10.1371/JOURNAL.PCBI.1007664. 32097405 PMC7059966

[19] M. I. Love , W. Huber , and S. Anders , “Moderated Estimation of Fold Change and Dispersion for RNA‐Seq Data With DESeq2,” Genome Biology 15, no. 12 (2014): 550, 10.1186/S13059-014-0550-8. 25516281 PMC4302049

[20] A. Alexa , J. Rahnenführer , and T. Lengauer , “Improved Scoring of Functional Groups From Gene Expression Data by Decorrelating GO Graph Structure,” Bioinformatics 22, no. 13 (2006): 1600–1607, 10.1093/BIOINFORMATICS/BTL140. 16606683

[21] J. Hadem , F. Tacke , T. Bruns , et al., “Etiologies and Outcomes of Acute Liver Failure in Germany,” Clinical Gastroenterology and Hepatology 10, no. 6 (2012): 664–669, 10.1016/j.cgh.2012.02.016. 22373724

[22] N. Weiler , A. Schlotmann , A. A. Schnitzbauer , S. Zeuzem , and M. W. Welker , “The Epidemiology of Acute Liver Failure,” Deutsches Ärzteblatt International 117, no. 4 (2020): 43–50, 10.3238/arztebl.2020.0043. 32036852 PMC7036472

[23] M. Villanueva‐Paz , L. Morán , N. López‐Alcántara , et al., “Oxidative Stress in Drug‐Induced Liver Injury (DILI): From Mechanisms to Biomarkers for Use in Clinical Practice,” Antioxidants 10 (2021): 390, 10.3390/antiox10030390. 33807700 PMC8000729

[24] G. C. Forcina and S. J. Dixon , “GPX4 at the Crossroads of Lipid Homeostasis and Ferroptosis,” Proteomics 19, no. 18 (2019): e1800311, 10.1002/pmic.201800311. 30888116

[25] D. U. Purcu , A. Korkmaz , S. Gunalp , et al., “Effect of Stimulation Time on the Expression of Human Macrophage Polarization Markers,” PLoS One 17, no. 3 (2022): e0265196, 10.1371/JOURNAL.PONE.0265196. 35286356 PMC8920204

[26] S. Desagher , A. Osen‐Sand , A. Nichols , et al., “Bid‐Induced Conformational Change of Bax Is Responsible for Mitochondrial Cytochrome c Release During Apoptosis,” Journal of Cell Biology 144, no. 5 (1999): 891–901, 10.1083/JCB.144.5.891. 10085289 PMC2148190

[27] C. S. Shao , X. H. Zhou , Y. H. Miao , P. Wang , Q. Q. Zhang , and Q. Huang , “In Situ Observation of Mitochondrial Biogenesis as the Early Event of Apoptosis,” iScience 24, no. 9 (2021): 103038, 10.1016/J.ISCI.2021.103038. 34553131 PMC8441175

[28] C. Mitchell , M. Mahrouf‐Yorgov , A. Mayeuf , et al., “Overexpression of Bcl‐2 in Hepatocytes Protects Against Injury but Does Not Attenuate Fibrosis in a Mouse Model of Chronic Cholestatic Liver Disease,” Laboratory Investigation 91, no. 2 (2011): 273–282, 10.1038/LABINVEST.2010.163. 20856227

[29] T. R. Knight , M. W. Fariss , A. Farhood , and H. Jaeschke , “Role of Lipid Peroxidation as a Mechanism of Liver Injury After Acetaminophen Overdose in Mice,” Toxicological Sciences 76, no. 1 (2003): 229–236, 10.1093/TOXSCI/KFG220. 12944590

[30] S. Dalaklioglu , G. E. Genc , N. H. Aksoy , F. Akcit , and S. Gumuslu , “Resveratrol Ameliorates Methotrexate‐Induced Hepatotoxicity in Rats via Inhibition of Lipid Peroxidation,” Human & Experimental Toxicology 32, no. 6 (2013): 662–671, 10.1177/0960327112468178. 23424212

[31] A. Gupta , V. Puri , R. Sharma , and S. Puri , “Folic Acid Induces Acute Renal Failure (ARF) by Enhancing Renal Prooxidant State,” Experimental and Toxicologic Pathology 64, no. 3 (2012): 225–232, 10.1016/J.ETP.2010.08.010. 20833517

[32] D. Martin‐Sanchez , O. Ruiz‐Andres , J. Poveda , et al., “Ferroptosis, but Not Necroptosis, Is Important in Nephrotoxic Folic Acid‐Induced AKI,” Journal of the American Society of Nephrology: JASN 28, no. 1 (2017): 218–229, 10.1681/ASN.2015121376. 27352622 PMC5198282

[33] Y. Ikeda , H. Hamano , Y. Horinouchi , et al., “Role of Ferroptosis in Cisplatin‐Induced Acute Nephrotoxicity in Mice,” Journal of Trace Elements in Medicine and Biology 67 (2021): 126798, 10.1016/J.JTEMB.2021.126798. 34087581

[34] Z. Hu , H. Zhang , B. Yi , et al., “VDR Activation Attenuate Cisplatin Induced AKI by Inhibiting Ferroptosis,” Cell Death & Disease 11, no. 1 (2020): 73, 10.1038/S41419-020-2256-Z. 31996668 PMC6989512

[35] C. Tang , M. J. Livingston , R. Safirstein , and Z. Dong , “Cisplatin Nephrotoxicity: New Insights and Therapeutic Implications,” Nature Reviews. Nephrology 19, no. 1 (2023): 53–72, 10.1038/S41581-022-00631-7. 36229672

[36] H. Qi , H. Shi , M. Yan , et al., “Ammonium Tetrathiomolybdate Relieves Oxidative Stress in Cisplatin‐Induced Acute Kidney Injury via NRF2 Signaling Pathway,” Cell Death Discovery 9, no. 1 (2023): 259, 10.1038/S41420-023-01564-1. 37491360 PMC10368633

[37] C. Yu , H. Dong , Q. Wang , et al., “Danshensu Attenuates Cisplatin‐Induced Nephrotoxicity Through Activation of Nrf2 Pathway and Inhibition of NF‐κB,” Biomedicine & Pharmacotherapy 142 (2021): 111995, 10.1016/J.BIOPHA.2021.111995. 34435595

[38] M. Dodson , M. R. de la Vega , A. B. Cholanians , C. J. Schmidlin , E. Chapman , and D. D. Zhang , “Modulating NRF2 in Disease: Timing Is Everything,” Annual Review of Pharmacology and Toxicology 59 (2019): 555–575, 10.1146/annurev-pharmtox-010818-021856. PMC6538038 30256716

[39] A. Kopacz , D. Kloska , H. J. Forman , A. Jozkowicz , and A. Grochot‐Przeczek , “Beyond Repression of Nrf2: An Update on Keap1,” Free Radical Biology & Medicine 157 (2020): 63–74, 10.1016/j.freeradbiomed.2020.03.023. 32234331 PMC7732858

[40] H. Tian , B. F. Zhang , J. H. Di , et al., “Keap1: One Stone Kills Three Birds Nrf2, IKKβ and Bcl‐2/Bcl‐xL,” Cancer Letters 325, no. 1 (2012): 26–34, 10.1016/j.canlet.2012.06.007. 22743616

[41] P. Ramadori , H. Drescher , S. Erschfeld , et al., “Hepatocyte‐Specific Keap1 Deletion Reduces Liver Steatosis but Not Inflammation During Non‐Alcoholic Steatohepatitis Development,” Free Radical Biology & Medicine 91 (2016): 114–126, 10.1016/j.freeradbiomed.2015.12.014. 26698665

[42] W. Bernal and J. Wendon , “Acute Liver Failure,” New England Journal of Medicine 369, no. 26 (2013): 2525–2534, 10.1136/pgmj.2004.026005. 24369077

[43] S. Tujios , R. T. Stravitz , and W. M. Lee , “Management of Acute Liver Failure: Update 2022,” Seminars in Liver Disease 42, no. 3 (2022): 362–378, 10.1055/s-0042-1755274. 36001996 PMC10576953

[44] N. Robledinos‐Antón , R. Fernández‐Ginés , G. Manda , and A. Cuadrado , “Activators and Inhibitors of NRF2: A Review of Their Potential for Clinical Development,” Oxidative Medicine and Cellular Longevity 2019 (2019): 9372182, 10.1155/2019/9372182. 31396308 PMC6664516

[45] R. N. Jadeja , K. K. Upadhyay , R. V. Devkar , and S. Khurana , “Naturally Occurring Nrf2 Activators: Potential in Treatment of Liver Injury,” Oxidative Medicine and Cellular Longevity 2016 (2016): 3453926, 10.1155/2016/3453926. 28101296 PMC5215260

[46] X. Li , J. Zhou , S. Chen , et al., “Role of Bicyclol in Preventing Chemotherapeutic Agent‐Induced Liver Injury in Patients Over 60 Years of Age With Cancer,” Journal of International Medical Research 42, no. 4 (2014): 906–914, 10.1177/0300060514527058. 24903556

[47] W. Naqiong , W. Liansheng , H. Zhanying , et al., “A Multicenter and Randomized Controlled Trial of Bicyclol in the Treatment of Statin‐Induced Liver Injury,” Medical Science Monitor 23 (2017): 5760–5766, 10.12659/MSM.904090. 29200411 PMC5728082

[48] S. Saha , N. Sachivkina , A. Karamyan , E. Novikova , and T. Chubenko , “Advances in Nrf2 Signaling Pathway by Targeted Nanostructured‐Based Drug Delivery Systems,” Biomedicine 12, no. 2 (2024): 403, 10.3390/biomedicines12020403. PMC10887079 38398005

[49] C. Knox , M. Wilson , C. M. Klinger , et al., “DrugBank 6.0: The DrugBank Knowledgebase for 2024,” Nucleic Acids Research 52, no. D1 (2024): D1265–D1275, 10.1093/nar/gkad976. 37953279 PMC10767804

[50] National Library of Medicine (US) . (2019, April 02 ‐ 2025, May 30) A Pharmacokinetic Study of Omaveloxolone in Subjects With Hepatic Impairment and Normal Hepatic Function. ClinicalTrials.gov identifier: NCT03902002. https://clinicaltrials.goc.study/NCT03902002.

