# Supplementary figures and images for: Keap1 Deletion Rescues Cell Death Associated With Gpx4 Loss in Hepatocytes During Acute Liver Injury
Source: Liver Int. 2025 Aug 22;45(9):e70210. doi: 10.1111/liv.70210 (PMC12372572; doi:10.1111/liv.70210)

**A**

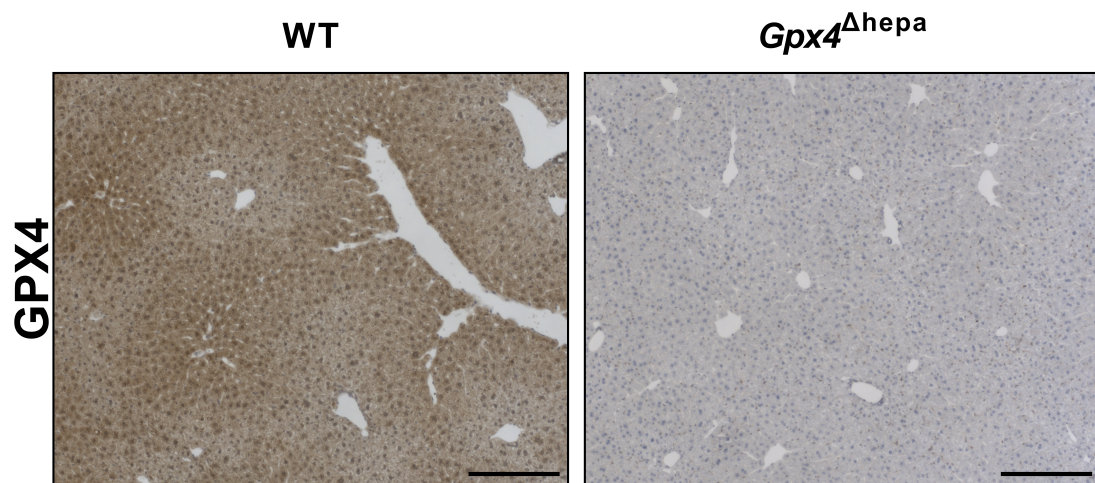

**B**

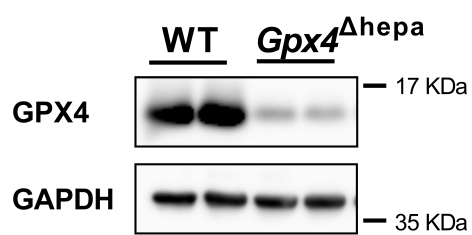

Supplement: Supplementary file 1 — Figure S1. Confirmation of GPX4 deletion in hepatocytes. (A) GPX4 IHC of WT (left) and Gpx4 Δhepa (right) mice. Scale bar = 100 μm. (B) Immunoblot analysis of liver extracts. GAPDH control shows equal loading of the protein in each line. [file LIV-45-0-s002.pdf]

**A**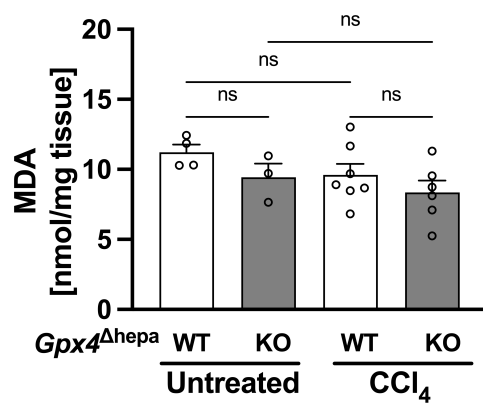**B**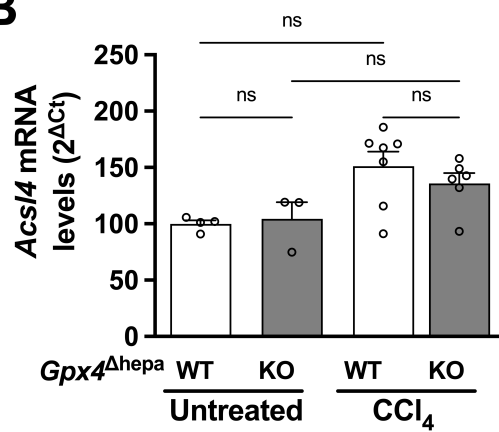**C**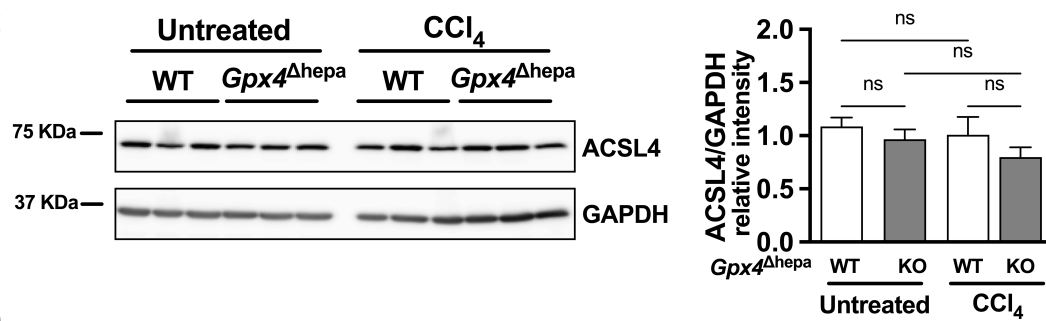**D**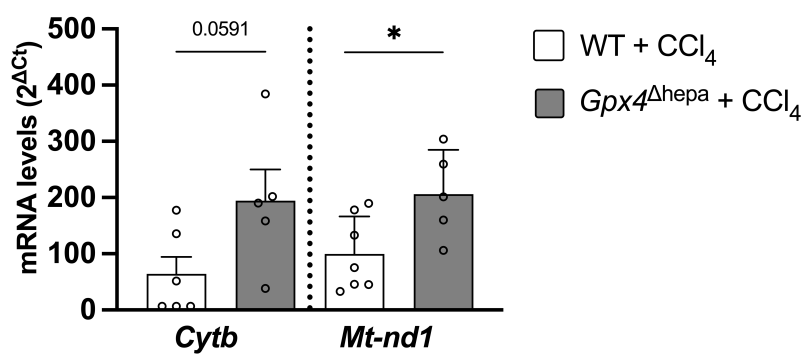

Supplement: Supplementary file 2 — Figure S2. Cell death mechanisms evaluation after CCl4 treatment. (A) MDA quantification in untreated and CCl4‐treated WT (untreated, n = 4; treated, n = 7; white) and Gpx4 Δhepa (untreated, n = 3, treated, n = 6; grey) mice. (B) RT‐qPCR of Acsl4 in untreated and CCl4‐treated WT (untreated, n = 4; treated, n = 7; white) and Gpx4 Δhepa (untreated, n = 3, treated, n = 6; grey) mice. (C) Left: immunoblot analysis of ACSL4. GAPDH control shows equal loading of the protein. Right: quantification of protein levels, normalised by GAPDH. D RT‐qPCR of mitochondrial biogenesis related genes Cytb and Mt‐nd1. Data are expressed as ± SEM; ns, not significant; * p < 0.05; ordinary one‐way ANOVA with Tukey's multiple comparison test was performed for panels A–C, unpaired t test comparing Gpx4 Δhepa to WT mice was conducted for panel D. Acyl‐CoA synthetase long chain family member 4, ACSL4; malondialdehyde, MDA; mitochondrially encoded cytochrome b, Cytb; mitochondrially encoded NADH:ubiquinone oxidoreductase core subunit 1, Mt‐nd. [file LIV-45-0-s006.pdf]

**A**

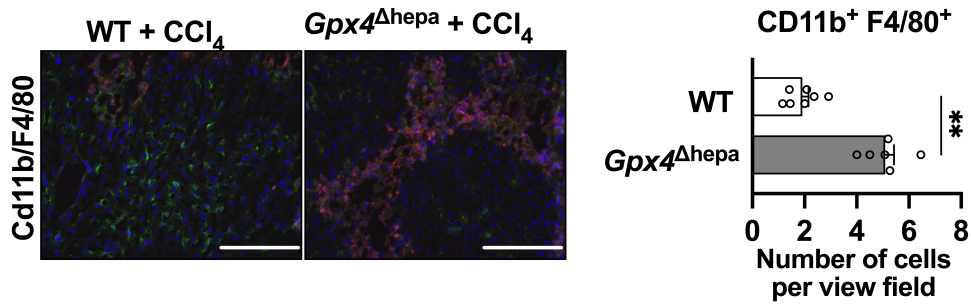

**B**

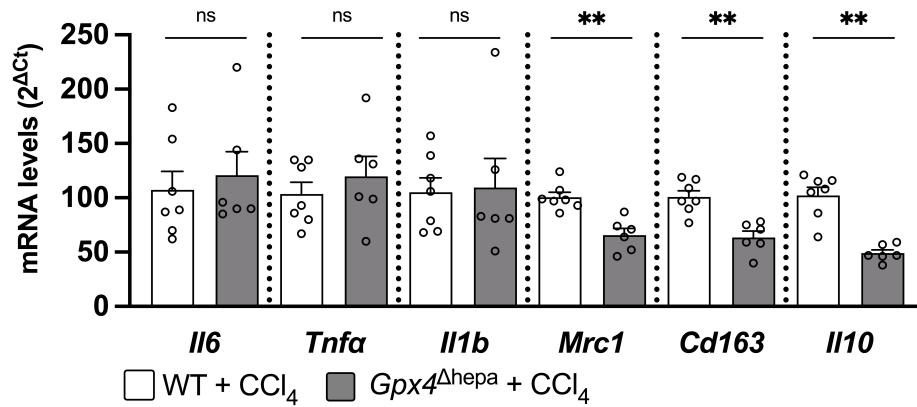

**C**

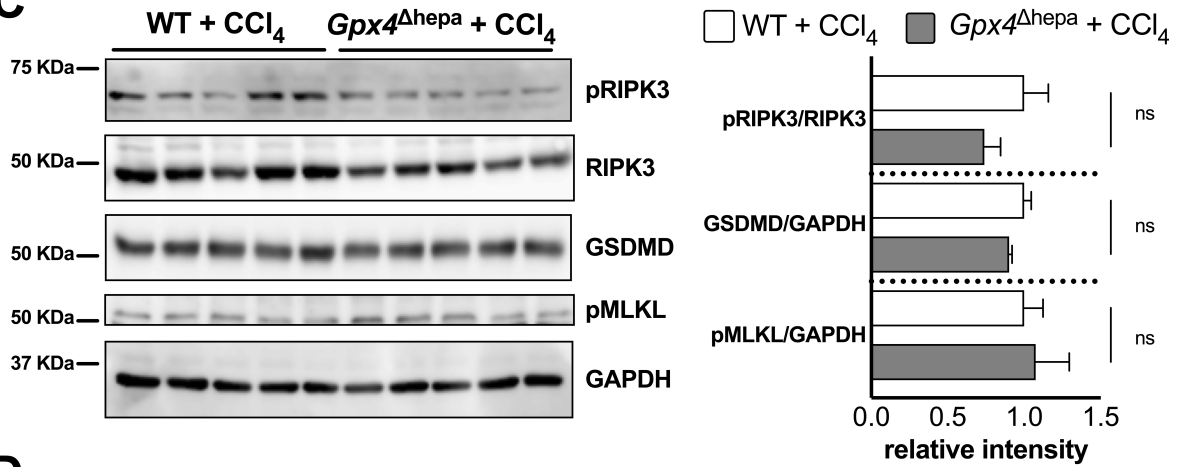

**D**

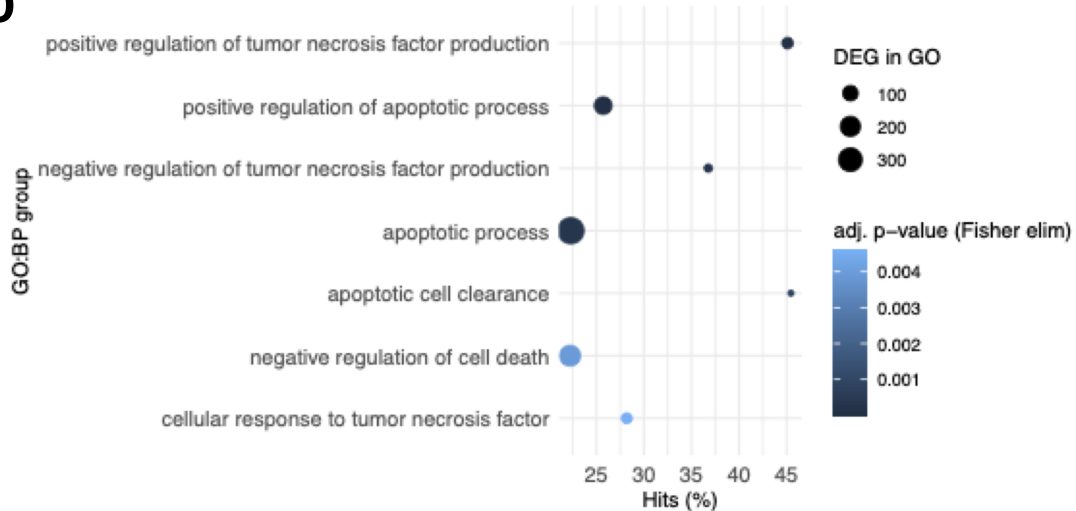

Supplement: Supplementary file 3 — Figure S3. Analysis of immune infiltration, cell death pathways and GSEA in CCl4‐treated mice. (A) Immunofluorescence staining of CD11b+F4/80+ macrophages in liver. Left: representative merged images with CD11b (magenta), F4/80 (green) and DAPI (blue) channels are shown. Scale bar = 200 μm. Right: quantification of cells co‐expressing CD11b and F4/80. (B) mRNA levels of macrophage polarisation markers (M1: Il6, Tnfα, Il1b; M2: Mrc1, Cd163, Il10) in WT and Gpx4 Δhepa treated with CCl4. Data are expressed as ± SEM from CCl4‐treated WT (n = 7) and Gpx4 Δhepa (n = 6). (C) Left: Immunoblot analysis of liver extracts for proteins involved in necroptosis (pRIPK3, RIPK3, and pMLKL) and pyroptosis (GSDMD). GAPDH control shows equal loading of the protein. Right: quantification of protein levels, normalised as indicated. D GO enrichment analysis of DEGs related to cell death in WT untreated versus WT + 24 h CCl4 mice. Data are expressed as ± SEM; ns, not significant; ** p < 0.01; unpaired t test comparing Gpx4 Δhepa to WT mice was conducted for panel A–C. CD163 molecule, Cd163; F4/80 glycoprotein, F4/80; gasdermin D, GSDMD; interleukin 1b, Il1b; interleukin 6, Il6; interleukin 10, Il10; integrin alpha M, CD11b; mannose receptor C‐type 1, Mrc1; phospho mixed lineage kinase domain like pseudokinase, pMLKL; (phospho‐) receptor‐interacting serine/threonine kinase 3, (p‐) RIPK3; tumour necrosis factor alpha, Tnfα. [file LIV-45-0-s004.pdf]

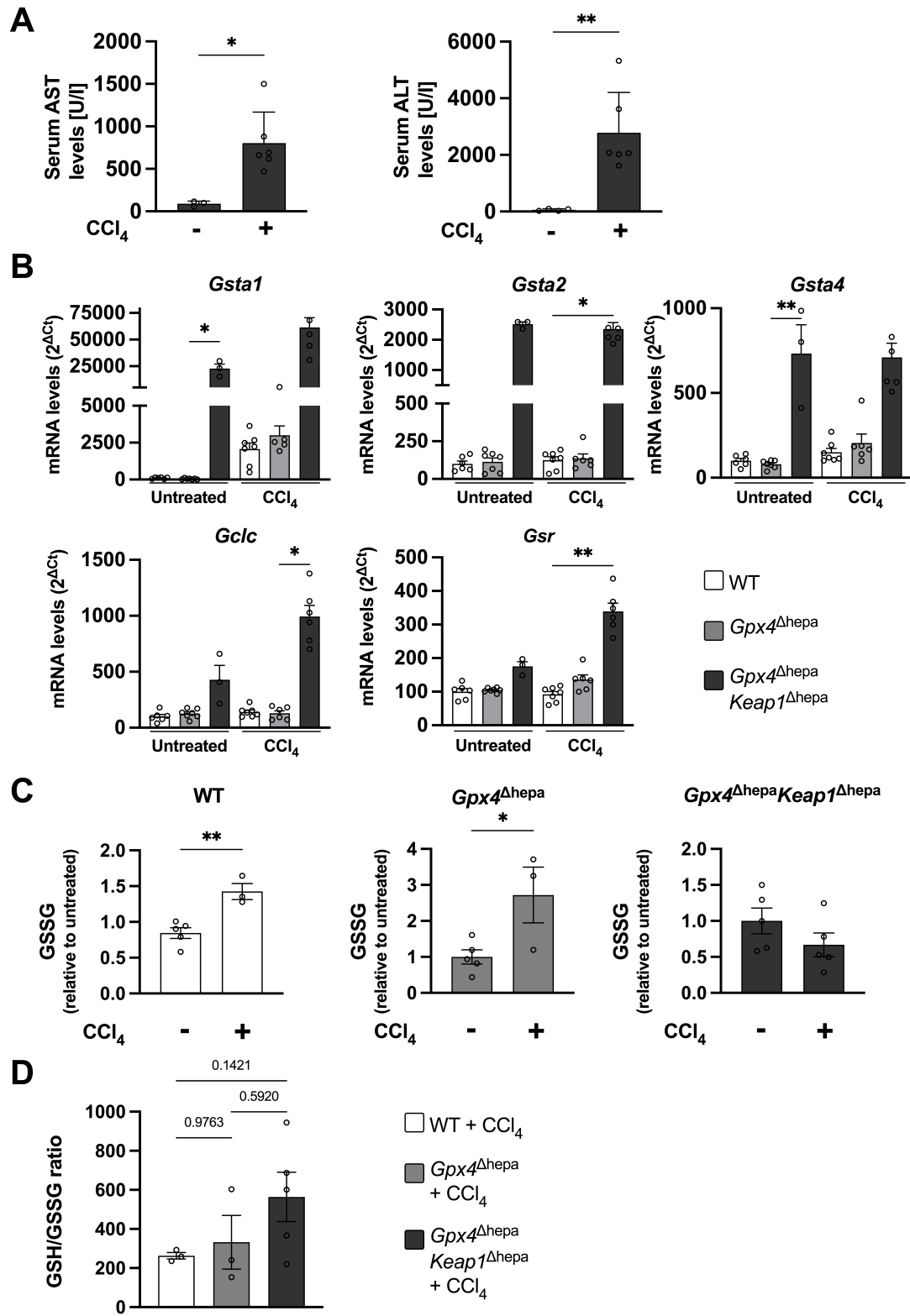

Supplement: Supplementary file 4 — Figure S4. Transaminases levels of untreated and CCl4‐treated Gpx4 Δhepa Keap1 Δhepa mice. (A) AST (left) and ALT (right) levels in serum from untreated (n = 4) and CCl4‐treated (n = 6) mice. (B) RT‐qPCR analysis for glutathione peroxidase‐active family members 1, 2 and 4 (above) and glutathione biosynthesis related genes (Gclc and Gsr) (below) from CCl4‐treated WT (n = 7; white), Gpx4 Δhepa (n = 6; grey), and Gpx4 Δhepa Keap1 Δhepa (n = 6; charcoal) mice. (C) GSSG quantification in CCl4‐treated versus untreated WT (untreated n = 5, CCl4 n = 3; white), Gpx4 Δhepa (untreated n = 5, CCl4 n = 3; grey), and Gpx4 Δhepa Keap1 Δhepa (untreated n = 5, CCl4 n = 5; charcoal) mice. D GSH/GSSG ratio in CCl4‐treated mice. Data are expressed as ± SEM; ns, not significant; * p < 0.05; ** p < 0.01; unpaired t test comparing treated to untreated Gpx4 Δhepa Keap1 Δhepa mice was conducted for panels A and C; for panels B and D ordinary one‐way analysis of variance (ANOVA) with Tukey's multiple comparison test was performed. Aspartate transaminase, AST; alanine transaminase, ALT; glutathione peroxidase‐active, Gsta; glutamate‐cysteine ligase catalytic subunit, Gclc; glutathione‐disulfide reductase, Gsr, oxidised form of glutathione, GSSG; reduced form of glutathione, GSH. [file LIV-45-0-s001.pdf]
