# Supplementary material for: Keap1 Deletion Rescues Cell Death Associated With Gpx4 Loss in Hepatocytes During Acute Liver Injury
Source: Liver Int. 2025 Aug 22;45(9):e70210. doi: 10.1111/liv.70210 (PMC12372572; doi:10.1111/liv.70210)
Supplement: Supplementary file 5 — Table S1. Etiologies associated with patients with liver failure. [file LIV-45-0-s003.docx]

**Supplementary Table 1.** Etiologies associated with patients with liver failure.

| **ALF** | |  | **ACLF** | |
| --- | --- | --- | --- | --- |
| **Etiology** | **Number of cases** |  | **Etiology** | **Number of cases** |
| Drug-induced hepatotoxicity | 8 |  | Hepatitis B virus reactivation | 3 |
| Acetominophen-induced hepatotoxicity | 1 |  | Drug-induced hepatotoxicity | 2 |
| Cryptogenic/unknown | 7 |  | Thrombosis | 2 |
| Total number of ALF patients = 16 | |  | Acute hepatitis E | 1 |
|  |  |  | Hemorrhage | 1 |
|  |  |  | Primary sclerosing cholangitis | 1 |
| Abbreviations: ACLF, acute-on-chronic liver failure; ALF, acute liver failure. | |  | Autoimmune hepatitis flare | 1 |
|  |  |  | Cryptogenic/unknown | 2 |
|  |  |  | Total number of ACLF patients = 14 | |
