# Supplementary material for: Keap1 Deletion Rescues Cell Death Associated With Gpx4 Loss in Hepatocytes During Acute Liver Injury
Source: Liver Int. 2025 Aug 22;45(9):e70210. doi: 10.1111/liv.70210 (PMC12372572; doi:10.1111/liv.70210)
Supplement: Supplementary file 6 — Table S2. Antibody list. [file LIV-45-0-s007.docx]

**Supplementary table 2.** Antibody list

| **Name** | **Supplier** | **Reference** | **Application** |
| --- | --- | --- | --- |
| 4HNE | Abcam | ab48506 | IHC (1:200) |
| ACSL4 | Abcam | ab155282 | WB (1:1000) |
| BCL2 | Cell Signaling Technology | 3498 | WB (1:1000) |
| CD11b PE | Thermo Fisher Scientiﬁc | 12-0112-82 | FC, myeloid |
| CD11b | Abcam | ab8878 | IF (1:200) |
| CD11c APC | BioLegend | 117310 | FC, myeloid |
| CD19 PerCP-Cy5.5 | BD | 551001 | FC, lymphoid |
| CD3e APC | Thermo Fisher Scientiﬁc | 12-0031-81 | FC, lymphoid |
| CD4 PE | Thermo Fisher Scientiﬁc | 12-0041-83 | FC, lymphoid |
| CD45 APC-Cy7 | BD | 557659 | FC, myeloid and lymphoid |
| CD8a FITC | Thermo Fisher Scientiﬁc | 11-0081-85 | FC, lymphoid |
| Cleaved caspase 3 | Cell Signaling Technology | 9661 | IHC (1:400) |
| F4/80 | Bio-Rad Laboratories | MCA497 | IF (1:200) |
| F4/80 PE-Cy7 | Thermo Fisher Scientiﬁc | 25-4801-82 | FC, myeloid |
| GAPDH | Bio-Rad Laboratories | MCA4739 | WB (1:5000) |
| γH2AX | Abcam | ab11174 | WB (1:1000) |
| Goat anti-rat/Alexa 488 | Invitrogen | A-11006 | IF (1:500) |
| Goat anti-rat/Cy3 | Invitrogen | A10522 | IF (1:5500) |
| GPX4 | Abcam | ab125066 | WB (1:1000), IHC (mouse, 1:500; human, 1:1600) |
| GSDMD | Cell Signaling Technology | 93709 | WB (1:1000) |
| HRP anti-mouse IgG | Santa Cruz Biotechnology | sc-516102 | WB (1:5000) |
| HRP anti-rabbit IgG | Cell Signaling Technology | 7074 | WB (1:5000) |
| KEAP1 | Cell Signaling Technology | 8047 | WB (1:1000) |
| Ly6C PerCP-Cy5.5 | Thermo Fisher Scientiﬁc | 45-5932-82 | FC, myeloid |
| Ly6G Alexa Fluor 700 | BioLegend | 127622 | FC, myeloid |
| MHCII FITC | BioLegend | 107605 | FC, myeloid |
| NK1.1 PE-Cy7 | Thermo Fisher Scientiﬁc | 25-5941-82 | FC, lymphoid |
| NRF2 | Proteintech Group | 16396-1-AP | WB (1:1000) |
| pMLKL | Abcam | ab196436 | WB (1:1000) |
| pRIPK3 | Cell Signaling Technology | 57220 | WB (1:1000) |
| RIPK3 | Cell Signaling Technology | 2283 | WB (1:1000) |

Abbreviations: FC, flow cytometry; IF, immunofluorescence; IHC, immunohistochemistry; WB, western blot.
