# Supplementary material for: Keap1 Deletion Rescues Cell Death Associated With Gpx4 Loss in Hepatocytes During Acute Liver Injury
Source: Liver Int. 2025 Aug 22;45(9):e70210. doi: 10.1111/liv.70210 (PMC12372572; doi:10.1111/liv.70210)
Supplement: Supplementary file 7 — Table S3. Primer list for RT‐qPCR. [file LIV-45-0-s005.docx]

**Supplementary table 3.** Primer list for RT-qPCR

| **Gene** | **Primer probe** | **Sequence** |
| --- | --- | --- |
| *Acsl4* | Forward | 5'-CGCTGTTCCGGAAATCATGG-3' |
|  | Reverse | 5'-GGGGCGTCATAGCCTTTCTT-3' |
| *Bad* | Forward | 5'-AGGTGGGGACGCCGGTAAGGAG-3' |
|  | Reverse | 5'-CTCCGGAAGTGGCTGTTTTC-3' |
| *Bax* | Forward | 5'-GAACCATCATGGGCTGGACA-3' |
|  | Reverse | 5'-GGAGAGGAGGCCTTCCCAG-3' |
| *Cd163* | Forward | 5'-TGCTGTCACTAACGCTCCTG-3' |
|  | Reverse | 5'-TCATTCATGCTCCAGCCGTT-3' |
| *Cd206* | Forward | 5'-CCCAAGGGCTCTTCTAAAGCA-3' |
|  | Reverse | 5'-ACGCCGGCACCTATCACA-3' |
| *Cyp2e1* | Forward | 5'-CTCCGCACGTCCTTCCATGTGG-3' |
|  | Reverse | 5'-GCTGTCAAGGAGGTGCTACTGAACC-3' |
| *Cytb* | Forward | 5'-GACCTCCCCACCCCATCCA-3' |
|  | Reverse | 5'-AAAGGCGGTTGAGGCGTCTG-3' |
| *Gapdh* | Forward | 5'-TCAAGCTCATTTCCTGGTATGAC-3' |
|  | Reverse | 5'-CTTGCTCAGTGTCCTTGCTG-3' |
| *Gclc* | Forward | 5'-TGCACATCTACCACGCAGTC-3' |
|  | Reverse | 5'-GTCTCAAGAACATCGCCTCCA-3' |
| *Gpx2* | Forward | 5'-CTCAGTGTACCCTCGGGAGA-3' |
|  | Reverse | 5'-TACCCCAGACTTAGAGCCCC-3' |
| *Gpx4* | Forward | 5'-ACCCACTGTGGAAATGGATGA-3' |
|  | Reverse | 5'-CTCTATCACCTGGGGCTCCTC-3' |
| *Gsr* | Forward | 5'-CACGACCATGATTCCAGATG-3' |
|  | Reverse | 5'-CAGCATAGACGCCTTTGACA-3' |
| *Gsta1* | Forward | 5'-CAGACCAGAGCCATTCTCAACTA-3' |
|  | Reverse | 5'-GGAGAACTTCCAGTAGGTGGAT-3' |
| *Gsta2* | Forward | 5'-AAGACTGCCTTGGCAAAAGA-3' |
|  | Reverse | 5'-GAGAAGAAGTTCCAGCAGGTG-3' |
| *Gsta4* | Forward | 5'-AGCCATTTTGATGGTGGAAG-3' |
|  | Reverse | 5'-CGGGTTGCAGGAACTTCTTA-3' |
| *Il10* | Forward | 5'-GGCGCTGTCATCGATTTCTC-3' |
|  | Reverse | 5'-CTCTTCACCTGCTCCACTGC-3' |
| *Il1b* | Forward | 5'-GCAGTGGTTCGAGGCCTAAT-3' |
|  | Reverse | 5'-CTCATCACTGTCAAAAGGTGGC-3' |
| *Il6* | Forward | 5'-ACTTCACAAGTCGGAGGCTT-3' |
|  | Reverse | 5'-TGCAAGTGCATCATCGTTGT-3' |
| *Mrc1* | Forward | 5'-ATGCTGTAGTACCGGAGGGT-3' |
|  | Reverse | 5'-CATGCCGTTTCCAGCCTTTC-3' |
| *Mt-nd1* | Forward | 5'-CAACCTCAACCTAGGCCTCCT-3' |
|  | Reverse | 5'-ACGGCTAGGCTAGAGGTGGC-3' |
| *Nqo1* | Forward | 5'-AGCCAATCAGCGTTCGGTAT-3' |
|  | Reverse | 5'-GCCTCCTTCATGGCGTAGTT-3' |
| *Pgd* | Forward | 5'-AACAAAGAGGCTTGGCCC-3' |
|  | Reverse | 5'-TCTTCAAATGCCTGAGCCA-3' |
| *Prdx6* | Forward | 5'-CCTGGAGCAAGGACATCAAT-3' |
|  | Reverse | 5'-GTTTCTTGTCAGGGCCAAAA-3' |
| *Sqstm1* | Forward | 5'-CCTTGCCCTACAGCTGAGTC-3' |
|  | Reverse | 5'-TTTCTGGGGTAGTGGGTGTC-3' |
| *Tnf* | Forward | 5'-ACTGAACTTCGGGGTGATCG-3' |
|  | Reverse | 5'-GCCATTTGGGAACTTCTCATCC-3' |
| *Txr* | Forward | 5'-ACTGCCAGGATGTTGCTG-3' |
|  | Reverse | 5'-TTCCTTGTTAGCACCGGAG-3' |
